# A Novel Homozygous Stop Mutation in *IL23R* Causes Mendelian Susceptibility to Mycobacterial Disease

**DOI:** 10.1007/s10875-022-01320-7

**Published:** 2022-07-13

**Authors:** Frederik Staels, Flaminia Lorenzetti, Kerstin De Keukeleere, Mathijs Willemsen, Margaux Gerbaux, Julika Neumann, Thomas Tousseyn, Emanuela Pasciuto, Paul De Munter, Xavier Bossuyt, Rik Gijsbers, Adrian Liston, Stephanie Humblet-Baron, Rik Schrijvers

**Affiliations:** 1 Department of Microbiology, Immunology and Transplantation, Laboratory of Adaptive Immunology, KU Leuven, Leuven, Belgium; 2 Department of Microbiology, Immunology and Transplantation, Allergy and Clinical Immunology Research Group, KU Leuven, Leuven, Belgium; 3 VIB Center for Brain and Disease Research, Leuven, Belgium; 4 Department of Imaging and Pathology, Laboratory for Translational Cell and Tissue Research, KU Leuven, Leuven, Belgium; 5 Department of General Internal Medicine, University Hospitals Leuven, Leuven, Belgium; 6 Department of Microbiology, Immunology and Transplantation, Laboratory of Clinical and Diagnostic Immunology, KU Leuven, Leuven, Belgium; 7 Department of Pharmaceutical and Pharmacological Sciences, Laboratory for Viral Vector Technology and Gene Therapy, KU Leuven, Leuven, Belgium; 8 Leuven Viral Vector Core, KU Leuven, Leuven, Belgium; 9 Laboratory of Lymphocyte Signaling and Development, Babraham Institute, Cambridge, UK; 10 Department of Neurosciences, Laboratory for the Research of Neurodegenerative Diseases, KU Leuven, Leuven, Belgium

**Keywords:** inborn errors of immunity, mendelian susceptibility to mycobacterial disease, IL23R, non-tuberculous mycobacteria, IFN-γ, th17

## Abstract

**Purpose:**

Mendelian susceptibility to mycobacterial disease (MSMD) is caused by inborn errors of IFN-γ immunity. The most frequent genetic defects are found in *IL12* or a subunit of its receptor. IL23R deficiency in MSMD has only been reported once, in two pediatric patients from the same kindred with isolated disseminated Bacille Calmette-Guérin disease. We evaluated the impact of a homozygous stop mutation in *IL23R* (R381X), identified by whole exome sequencing, in an adult patient with disseminated non-tuberculous mycobacterial disease.

**Methods:**

We performed functional validation of the R381X mutation by evaluating IL23R expression and IL-23 signaling (STAT3 phosphorylation, IFN-γ production) in primary cells (PBMCs, EBV-B cells) and cell lines (HeLa) with or without back-complementation of wild-type IL23R.

**Results:**

We report on a 48-year-old male with disseminated non-tuberculous mycobacterial disease. We identified and characterized a homozygous loss-of-function stop mutation underlying IL23R deficiency, resulting in near absent expression of membrane bound IL23R. IL23R deficiency was characterized by impaired IL-23-mediated IFN-γ secretion in CD4^+^, CD8^+^ T, and mucosal-associated invariant T (MAIT) cells, and low frequencies of circulating Th17 (CD3^+^CD45RA^−^CCR4^+^CXCR3^−^RORγT^+^), Th1* (CD45RA^−^CCR4^−^CXCR3^+^RORγT^+^), and MAIT (CD3^+^CD8^+^Vα7.2^+^CD161^+^) cells. Although the patient did not have a history of recurrent fungal infections, impaired Th17 differentiation and blunted IL-23-mediated IL-17 secretion in PBMCs were observed.

**Conclusion:**

We demonstrate that impaired IL-23 immunity caused by a homozygous R381X mutation in *IL23R* underlies MSMD, corroborating earlier findings with a homozygous p.C115Y IL23R mutation. Our report further supports a model of redundant contribution of IL-23- to IL-17-mediated anti-fungal immunity.1

**Supplementary Information:**

The online version contains supplementary material available at 10.1007/s10875-022-01320-7.

## Introduction

Mendelian susceptibility to mycobacterial disease (MSMD) is caused by inborn errors of IFN-γ immunity [1–3]. Patients with MSMD are defined as individuals who are susceptible to infections by weakly virulent environmental mycobacteria and Bacillus Calmette-Guérin (BCG) disease post vaccination [4]. To date, 19 genes are implicated in MSMD (*CYBB, IFNGR1, IFNGR2, IFNG, IL12RB1, IL12B, IL23R, IL12RB2, ISG15, IRF8, JAK1, NEMO, RORC, SPPL2A, STAT1, TBX21, TYK2, USP18, ZNFX1)*. The different genetic disorders are further defined by the nature of the causal alleles (null or hypomorphic), protein levels (normal, low, or absent), and the mode of inheritance (autosomal recessive: AR, autosomal dominant: AD, or X-linked recessive: XL) and whether they present as isolated MSMD or as a part of a broader spectrum (syndromic MSMD) involving infectious susceptibility to other pathogens and/or autoinflammation [1, 5, 6]. The most frequent genetic defects are found in *IL12* or a subunit of its receptor (AR complete IL12p40 deficiency or AR IL12RB1 deficiency), followed by defects in the IFN-γ receptor (AD or AR complete/partial IFNGR1/2 deficiency) [1]. For other genes such as *IL23R* or *IL12RB2*, only a single kindred with functional validation of the corresponding genetic defect has been described [7]. This is not hypothesized to be due to increased rarity of loss of function (LOF) mutations in these genes, but due to the lower penetrance of these deficiencies [7]. In case of IL23R deficiency, two affected pediatric-onset cases from the same kindred with a phenotype of disseminated BCG disease were found to have a pathogenic homozygous mutation (p.C115Y) in the extracellular domain of IL23R. Although the mutation did not impair mRNA or protein expression, reduced membrane-bound IL23R and impaired IL-23 signaling was observed. Another IL23R mutation (p.R381Q) has been reported as a partial LOF variant and associates with pulmonary tuberculosis severity [8]. In this study, we describe a kindred with a homozygous mutation (c.1141C > T, R381X) in the intracellular domain of IL23R, presenting as adult-onset disseminated non-tuberculous mycobacterial disease. We used in silico prediction tools, immunophenotyping, and functional analysis of peripheral blood mononuclear cells (PBMCs) and cell lines to corroborate the genotype–phenotype relation in this patient.

## Methods

### gDNA Extraction and Whole Exome Sequencing

For gDNA extraction from whole blood, Purelink DNA Genomic DNA Mini kit (ThermoFisher Scientific) was used according to the manufacturers’ instructions. Whole exome sequencing was performed using SureSelect Human All Exon V7 (Agilent) for exome capture (Macrogen®, the Netherlands).

### PBMC Isolation

Whole blood was diluted 1:1 with RPMI 1640 and layered over lymphocyte separation medium (LSM) (MP Biomedicals, 0,850,494-CF). Tubes were centrifuged at 400 G for 25 min and PBMCs were harvested and stored in liquid nitrogen until FACS staining or used immediately for functional assays.

### mRNA Isolation and Synthesis of cDNA

mRNA was isolated from PBMCs using TRIzol reagent (Life Technologies). Five hundred microliters of TRIzol was added to PBMCs and frozen at − 80 °C. Later, samples were thawed and mRNA isolated according to manufacturer instructions. Complementary DNA (cDNA) was synthesized from RNA using the GoScript™ Reverse Transcription System (Promega) according to manufacturer instructions.

### qPCR

Forward and reverse primers were purchased from Integrated DNA Technologies (IDT). A Taqman probe was designed on exon 2 (ccagacatgaatcaggtcactattcaatgg) with primers located on the span junction of exon1-2 (forward primer tcaaacaggttgaaagagggaaac) and exon 2–3 (reverse primer tcctccatgacaccagctga) and a Taqman probe on exon 8 (tgatcgtctttgctgttatgttgtcaattctttct) with primers on the span junction of exon 7–8 (forward primer acagggcaccttacttctgacaac) and on exon 8 (reverse primer agttcggaatgatctgttaaatatccc) HPRT1 or GAPDH was used as housekeeping gene for all performed qPCRs. The reaction was performed as followed: 0.6 µl of primers (0.30 µM), 0.4 µl of probe (0.25 µM), 6 ng of DNA (2 ng/µL) and 1 × Taqman Fast mastermix (ThermoFisher Scientific) were mixed and plated on a 96-well plate. The plate was run on the StepOneTM Real-Time PCR system (ThermoFisher Scientific) and analyzed with StepOne Software v2.3.

### Stimulation

STAT3 and 4 phosphorylation was assessed by flow cytometry and Western blot after stimulation. For stimulation, fresh PBMCs or EBV-transformed B cells were plated overnight, and stimulated for 30 min with IL-23 (R&D 1290-IL, 10 or 100 ng/mL), IL-6 (R&D 206-IL, 10 ng/mL), IFN-α2b (Invivogen rcyc-hifna2b, 10 ng/mL), IL-12 (Biolegend 573,002, 40 ng/mL) in complete RPMI 1640 medium (cRMPI: supplemented with penicillin–streptomycin, FBS 5% + HEPES + MEM non-essential aminoacids) (GibcoTM). For STAT4-phosphorylation, PBMCs were seeded at 2 × 10^5^ cells/mL in a 48-well plate and preactivated with plate-bound anti-CD3 antibody (16–0038-85, Invitrogen, 10 µg/ml) and soluble anti-CD28 antibody (16–0289-85, Invitrogen, 5 µg/ml) for 3 days, before stimulation with IL-12.

### Western Blot

PBMCs or EBV-B cells were lysed in lysis buffer (50 mM Tris–HCl pH 7.5, 135 mM NaCl, 1.5 mM MgCl2, 1% Triton-X, 10% glycerol, 1 × protease inhibitor (Pierce TM Protease Inhibitor, ThermoFisher Scientific) and 1 × phosphatase inhibitor (PhosSTOP, Roche)). Protein concentrations were determined using a Bradford Protein Assay (Bio-Rad). Protein lysate was denaturized in LDS (NuPAGE LDS sample buffer, Novex) and DTT (Bio-Rad) at 70 °C for 10 min and was then loaded on a 4–12% Bis–Tris polyacrylamide gel (BoltTM Bis–Tris Plus, Thermo Fisher Scientific) in MOPS buffer (NuPAGE MOPS SDS running buffer, Novex). Separated proteins on the gel were transferred onto a methanol-activated PVDF membrane (GE Healthcare, Little Chalfont, UK) in transfer buffer (10% methanol, 1 × Tris/Glycine Buffer (Bio-Rad)) using the Tetra Blotting Module (Bio-Rad). The PVDF membrane was blocked in 5% milk Tris-buffered saline with Tween 0.1% (TBS-T) for 30 min at RT and then incubated with primary antibody O/N at 4 °C followed by a wash with TBS-T and incubation with a secondary antibody for 1 h at RT. Primary antibodies used for Western blot: STAT3 (9132, CST), pSTAT3 (Y705, 9145, CST) STAT4 (2653, CST), p-STAT4 (5267, CST) and mouse anti-GAPDH (Invitrogen). Secondary antibodies used were conjugated with HRP: goat anti-rabbit (abcam) and anti-mouse (Rockland). Secondary antibodies were visualized using ECLTM15 Prime Western Blotting Detection Reagent (AmershamTM) with the G:box Chemi-XRQ and quantified using ImageJ.

### Cloning

pWPXL_EF1a_hIL23R-3HA_Ires_eGFP was generated using Gibson Assembly (NEB). First, hIL23R-3HA gene sequence was PCR amplified from the pCH_SFFV_hIL23R_3HA_tCD34 plasmid (from Leuven Viral Vector Core and Molecular Virology and Gene Therapy Laboratory at the KU Leuven, BE) using primers: FW: 5’- acgggatccaggcctaagcttacgcgtgccaccatgaatcaggtcact-3’, RV: 5’-agtcgactcatatatcggaattcctaggcataatcaggcacgtcata-3’. Next, the amplified hIL23R-3HA was inserted into pWPXL_EF1a_Ires_eGFP transfer plasmid, previously digested with EcoRI and MluI restriction enzymes. pWPXL_EF1a_hIL23R-3HA_IRES_eGFP plasmid was transformed into DH5a bacterial competent cells and plasmids extracted by midi-prep (Macherey–Nagel). For IL12RB1 and IL23R (F380-HA) a commercial plasmid (cat nr. SC1200 and OHU25802D) was bought from GeneScript in a pcDNA3.1 vector containing respectively a C-terminal Myc or HA tag.

### Mutagenesis

pWPXL_EF1a_hIL23R-3HA_IRES_eGFP was mutagenized to insert the studied hIL23R variants (p.R381Q and p.R381X). pWPXL_EF1a_hIL23R-3HA_IRES_eGFP was PCR amplified by Q5 high fidelity DNA polymerase (NEB) using the following primers set: FW: 5’- atttaacagatcattccaaactgggattaaaag-3’, RV: 5’-cttttaatcccagtttggaatgatctgttaaat-3’ (p.R381Q). FW: 5’-atttaacagatcattctgaactgggattaaaag-3’, RV:5’- cttttaatcccagttcagaatgatctgttaaat-3’ (p.R381X). KLD enzyme Mix (NEB) was used to degrade the DNA template by DpnI. The mutagenized plasmids were transformed into DH5a bacterial competent cells and plasmids extracted by midi-prep (Macherey–Nagel).

### Transfection of HeLa and HEK293T Cells

HeLa or HEK293T cells were seeded in a 6-well plate at a density of 1 × 10^5^ cells/well in DMEM medium supplemented with FBS 5%, HEPES 25 mM and MEM non-essential amino acids) (GibcoTM). When reaching 70% confluency, transfection of C terminal HA tagged plasmids (empty vector, IL23R wild type (WT), IL23R-R381Q, IL23R-R381X, IL23R-F380) was performed using Lipofectamine 3000 (ThermoFisher Scientific) according to the manufacturer’s protocol. After 4 h, the medium was changed and cells were rested overnight. Subsequently, HeLa cells were stimulated with IL-23 (R&D 1290-IL, 100 ng/mL) or IFN-α2b (Invivogen rcyc-hifna2b, 10 ng/mL) for 30 min. Afterwards, cell lysis, protein extraction, quantification, and Western blot were performed as described before (section Western Blot for STAT3 phosphorylation).

### Confocal Microscopy

HeLa cells were seeded in a 48-well plate at a density of 1 × 10^4^ cells/well on a 12-mm coverslip precoated with poly-D-lysine (Sigma). Transfection was performed as described above. Coverslips were washed in PBS 1 × and fixed with paraformaldehyde 4% for 20 min. Afterwards, they were permeabilized (Triton 0.2% in PBS 1 ×), blocked (BSA 2%, Triton 0.2%), and stained with anti-HA (26,183, ThermoFisher Scientific), anti-Myc (71D10, CST), anti-GFP (A-21311 AF488 conjugated, ThermoFisher Scientific) in dilution buffer (BSA 1%, Triton 0.1%) overnight. Afterwards, coverslips were washed (Triton 0.05% in PBS 1 ×) and stained for 1 h with secondary antibodies (AF647 anti-rabbit (ab150083, Abcam) and AF568 anti-mouse (ab175473, Abcam)). Subsequently, coverslips were washed. Images were acquired using a Nikon AXR confocal unit, with a 60 × NA 1.4 oil objective at a 2048 × 2048 pixels resolution.

### Lentiviral Vector Production

Lentiviral vectors LV.EF1a:hIL23Rwt-3HA-IRES-eGFP and LV.EF1a: eGFP were generated by co-transfection of HEK293T cells with p.sPAX2, p.MD2.G-VSV.G and the transfer plasmids pWPXL_EF1a_hIL23Rwt-3HA_Ires_eGFP and pWPXL_EF1a_ eGFP by using X-tremeGENE HP Transfection Reagent (Sigma-Aldrich). pMD2.G and psPAX2 were a gift from Didier Trono (Addgene plasmid #12,259 and #12,260).

### Lentiviral Transduction

EBV-B cells were plated in a 96-well plate at a seeding density of 1 × 10^5^ cells in 200 μL of cRPMI and transduced using LV vectors as described above. After adding the lentiviral vectors, cells were spinoculated for 90 min at 35 °C and 800 G. Afterwards, they were resuspended and medium was changed at day 1 and 3. At day 4, cells were centrifuged and resuspended in stimulation medium (IL-23 100 ng/mL, IFN-α 10 ng/mL) for 60 min. Subsequently, cells were lysed and Western blot was performed as described above (section Western Blot for STAT3 phosphorylation).

### Staining

#### STAT3 and 4 Phosphorylation Assays

PBMCs were stained with fixable viability dye Zombie Aqua at room temperature for 10 min, washed twice in FACS buffer (3% FBS in PBS1 ×) followed by staining for the following surface markers before fixation and permeabilization: CD3 (11–0036-42, Invitrogen, FITC), CD4 (25–0047-42 or 47–0049-42, Invitrogen, PE-Cy7 or APC-Cy7), CD8 (17–0086-42 or 25–0088-42, Invitrogen, APC or PE-Cy7). Cells were fixed with BD cytofix for 10 min at 37 °C. Afterwards, permeabilization was performed using BD phosflow Perm III (558,050, BD Biosciences) on ice for 30 min followed by intracellular staining with p-STAT3 (560,312, BD Biosciences, Pacific Blue, pY705) or p-STAT4 (17,186,668, ThermoScientific, APC, pY693) for 60 min at 4 °C.

#### Th Compartment

Frozen PBMCs were thawed and counted, and cell concentration was adjusted to 5 × 10^6^ for each sample. Cells were plated in a V-bottom 96-well plate, washed once with PBS (Fisher Scientific), and stained with live/dead marker and fluorochrome-conjugated antibodies recognizing surface markers: anti-CD8-BUV805 (clone SK1), anti-CD4-BUV496 (clone SK3), anti-CD95-BUV737 (clone DX2), anti-CD28-BB660-P (clone CD28.2), anti-ICOS-BB630 (clone DX29), anti-CXCR3-BV785 (clone 1C6), anti-PD-1-BV750-P (clone EH12.1), anti-CXCR5-BV650 (clone RF8B2), anti-CCR2-BV605 (clone 1D9) anti-CD31-BV480 (clone WM59), (all BD Biosciences); anti-CD3-PerCP-Vio700 (clone REA613) (Miltenyi Biotec); anti-CD45RA-FITC (clone HI100), anti-CD14-PE-Cy5.5 (clone TuK4), anti-CCR7-PE-Cy7 (clone 3D12) (all eBioscience); anti-CD25-BV711 (clone BC96), anti-HLA-DR-BV570 (clone L243), anti-CD127-BV421 (clone A019D5), anti-CCR4-PE/Dazzle 594 (clone L291H4) (all BioLegend). Samples were stained for 60 min at 4° C, washed twice in PBS/1% FBS (Tico Europe), and then fixed and permeabilized with Foxp3 Transcription Factor Staining Buffer Set (eBioscience), according to manufacturer’s instructions. Cells were stained overnight at 4° C with anti-Ki67-BUV615-P (clone B56), anti-CTLA-4-PE-Cy5 (clone BNI3), anti-RORγt-PE (clone Q21-559) (all BD Biosciences), and anti-FOXP3-AF647 (clone 206D) (BioLegend) anti-human intracellular antibody, and were then acquired on a Symphony flow cytometer with Diva software (BD Biosciences). A minimum of 5 × 10^5^ events were acquired for each sample. Compensation Beads (ThermoFisher Scientific) were used to optimize Fluorescence compensation settings for multi-color flow cytometric analysis at a Symphony flow cytometer.

#### IL23R

PBMCs were isolated as described above and plated overnight. On the next day, cells were washed in PBS 1 × and stained for 30 min with anti-CD3 (17–0038-42, APC-Cy7), anti-CD4 (Pacific Blue, anti-CD8 (25–0088-42, PE-Cy7) (all Invitrogen), IL23R (ab222104, Abcam), and Zombie Aqua fixable viability dye. Cells were washed in FACS buffer (FBS 3% in PBS 1 ×) and stained for 30 min with secondary AF647 anti-rabbit (ab150083, Abcam). Data acquisition was performed using the BD FACSCanto II and analyzed using FlowJo software.

##### Th17 Differentiation

Naïve T cells were isolated from PBMCs by magnetic purification (MAGH115, R&D). They were cultured in Th0 conditions (anti-CD3 10 µg/mL, anti-CD28 5 µg/mL) or Th17 (anti-CD3 10 µg/mL, anti-CD28 5 µg/mL, TGF-β 10 ng/mL (Peprotech), IL-1β 10 ng/mL (Peprotech), IL-23 10 ng/mL (R&D 1290-IL), IL-6 10 ng/mL (Peprotech), anti-IL-4 µg/mL, anti-IFNγ 20 µg/mL) for 7 days. Supernatant was used for measurement of IL-17 by ELISA according to the manufacturer’s instructions (DY317-05, R&D).

##### IFN-γ Measurement

IFN-γ was assessed by flow cytometry and ELISA after stimulation. For alpha beta T cell subset and MAIT stimulation, fresh PBMCs were plated on an anti-CD3 (Invitrogen, 16–0038-85, 10 µg/mL) coated 48-well plate at a density of 2 × 10^5^ cells/mL with addition of soluble anti-CD28 (Invitrogen, 16–0289-85, 5 µg/mL) in RPMI 1640 medium (supplemented with penicillin–streptomycin, FBS 5% + HEPES 25 mM + MEM non-essential amino acids) (GibcoTM). At day 1 of culture, IL-23 (R&D 1290-IL, 100 ng/mL) and IL-12 (Biolegend, 573,002, 40 ng/mL) were added. At day 7, Brefeldin A (Abcam, 5 µg/mL) was added for 4 h to the culture to assess intracellular cytokine production by flow cytometry. For NK and iNKT cells, fresh PBMCs were stimulated for 48 h with IL-23 or IL-12 and Brefeldin A was added during the last 8 h of stimulation. In brief, cells were washed with PBS 1 × , stained with fixable viability dye Zombie Aqua at room temperature for 10 min, washed twice in FACS buffer followed by staining for surface markers: anti-CD3 (11–0036-42, Invitrogen, FITC), anti-CD4 ( Pacific Blue), anti-CD8 (25–0088-42, Invitrogen, PE-Cy7), anti-Vα7.2 (351,708, Biolegend, APC), anti-CD161 (45–1619-42, Invitrogen, PerCP Cy5.5), anti-CD56 (35–0567-42, eBioscience, PE Cy5.5) for 30 min at 4 °C. Later, cells were permeabilized using eBio Fix/Perm, washed twice in Permeabilization buffer, and stained with anti-IFN-γ (47–7319-42, ThermoFisher Scientific, APC-e780) for 30 min at 4 °C. Data acquisition was performed using the BD FACSCanto II and analyzed using FlowJo software. For mycobacterial stimulation, cells were plated in the same medium, left unstimulated or co-cultured with *Mycobacterium abscessus* strain (ATCC19977) (MOI 2) in the presence or absence of IL-23 (R&D 1290-IL, 100 ng/mL) or IL-12 (Biolegend, 573,002, 40 ng/mL) for 3 or 7 days. As a positive control, PMA/ionomycin (25 ng/mL + 1 µg/mL) without mycobacteria was used. Supernatant was used to measure IFN-γ by ELISA according to the manufacturer’s instruction (DY285B, R&D).

### Statistical Analysis

Statistical analysis was only performed if three biological replicates were available in each group. To compare between healthy controls and patient, an unpaired Student *t*-test (if normally distributed by Shapiro–Wilk) or Mann–Whitney *U* test (no normal distribution) was used. To compare the effects of stimulation in the healthy control or patient group, a paired Student *T* test, one-way ANOVA, or a Friedman test with post hoc tests was used depending on normality of the data. Significance levels were defined as followed: *p < 0.05, **p < 0.01, ***p < 0.001. Bars represent the standard error of the mean (SEM).

## Results

### Identification of a Homozygous R381X IL23R Mutation in a Kindred with MSMD

**Fig. 1:**
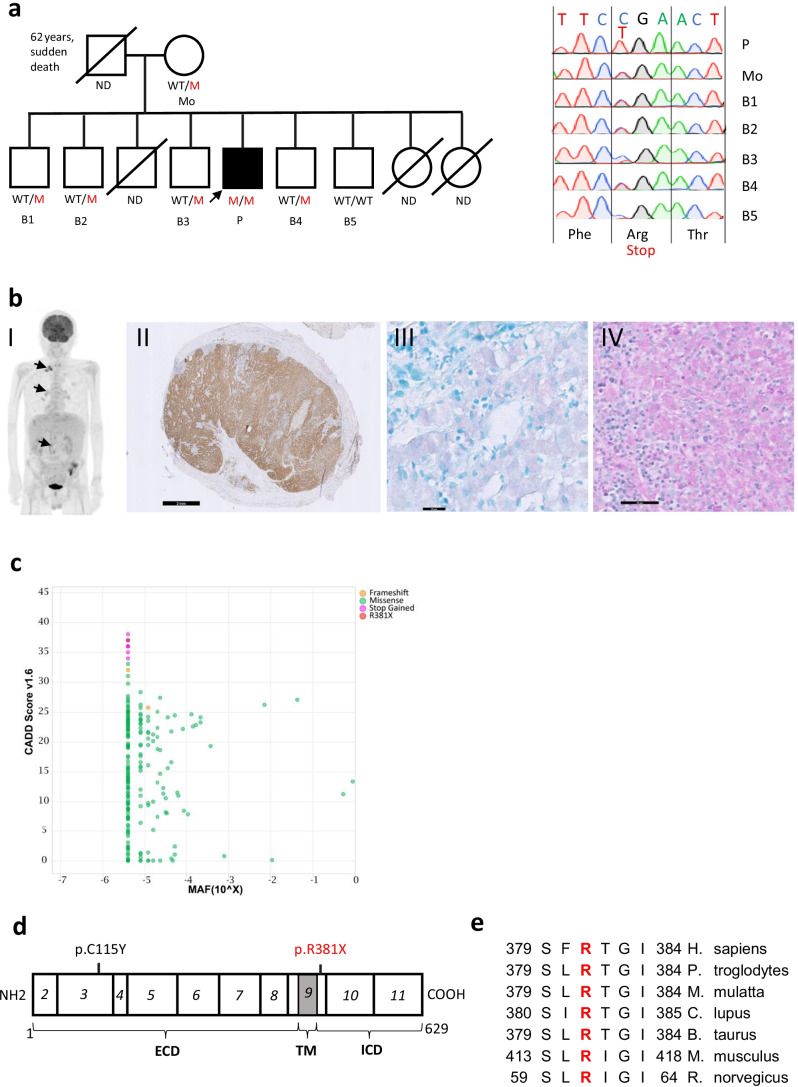
Identification of a homozygous c.1141C > T (p.R381X) in a patient with MSMD. **a** Family pedigree with genotype. ND = not determined. M = mutant. WT = wild type. Sanger chromatogram shown for all tested individuals. **b** b.I clinical presentation with multifocal adenopathies on PET CT scan resulting from disseminated nontuberculous mycobacterial disease, b.II CD68 staining showing diffuse histiocytic infiltration in a lymph node, b.III Positive Ziehl–Neelsen staining in macrophages on a duodenal biopsy, b.IV Periodic acid-Schiff (PAS) staining on bone marrow highlighting numerous PAS-positive micro-organismas within macrophage. Scale bars are depicted in black (2 mm, 20 µm and 50 µm). **c** Combined Annotation Dependent Depletion (CADD) score and allelic frequency (MAF) of the c.1141C > T (p.R381X) mutation and other predicted LOF heterozygous mutations in gnomAD. **d** Schematic linear protein structure of IL23R (629 aa) and exons (italic) encoding the extracellular domain (ECD), transmembranous domain (TM) and intracellular domain (ICD). The studied mutation (p.R381X) is indicated in red, a previously identified pathogenic mutation (p.C115Y) in black. **e** conservation of the R381 residue among mammalian species

A 48-year-old patient from Turkish descent (Fig. 1a) was diagnosed at the age of 41 years with a disseminated multi-mycobacterial infection, with pulmonary (*Mycobacterium avium* complex, culture, and PCR proven), bone marrow (presence of acid-fast bacilli, not identified by culture nor PCR), and gastrointestinal (*Mycobacterium tilburgii*, PCR proven) manifestations (Fig. 1b). Despite tuberculostatic treatment during 10 months, he suffered from protracted febrile episodes, systemic inflammation, and general weakness (for detailed case description see supplementary data). Using whole exome sequencing, a homozygous stop mutation in *IL23R* (p.R381X) was found in the patient and in heterozygous state in his mother and all of his siblings except for one. He had one brother who died of lung cancer and two sisters who died at an early age (no genetic material available), although information on the cause of death could not be retrieved. The other siblings were healthy. The R381X mutation was reported once in heterozygosity in the healthy population database but was not found in homozygosity (gnomAD v2.1.1, ExAC, 1000 Genomes). In silico tools (9) predict the mutation to be deleterious (Fig. 1c, CADD 37) and it resides in the intracellular domain of the IL23R (Fig. 1d), before the JAK2 binding site which is necessary for further downstream signaling through STAT3. This residue is well conserved among mammalian species (Fig. 1e).

### R381X Results in Near Absence of Membrane-Bound IL23R

**Fig. 2:**
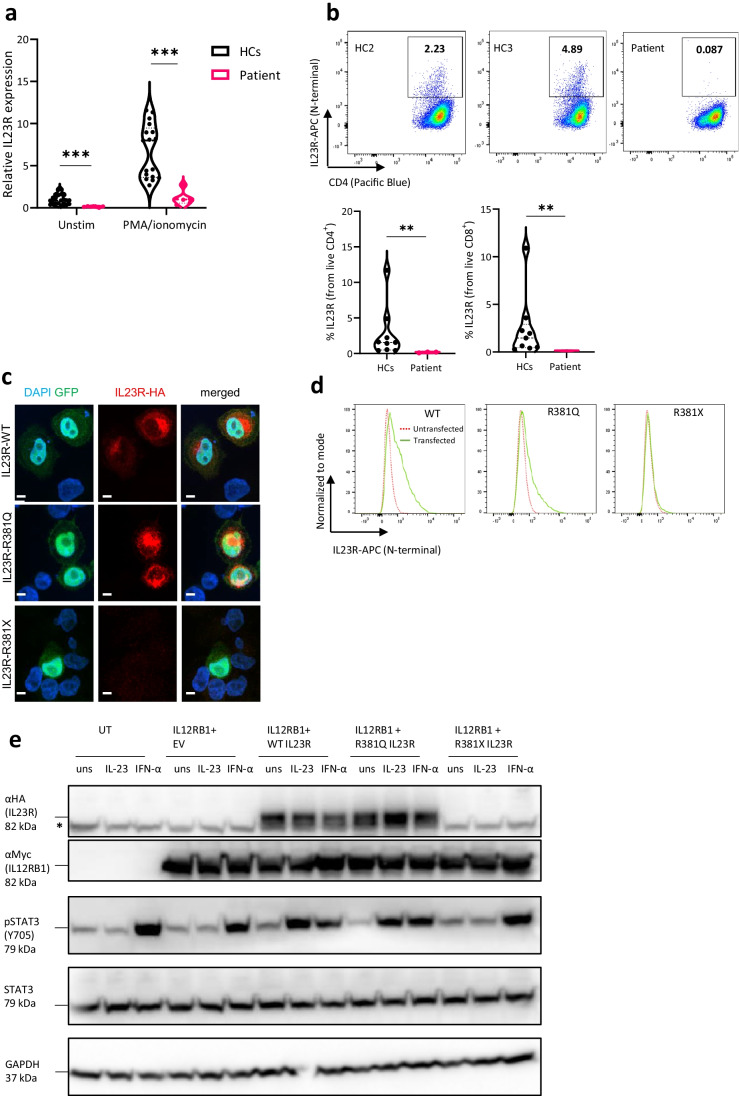
Near absence of IL23R expression in patient PBMCs and absent activity of overexpressed IL23R R381X in HeLa cells. **a** qPCR for *IL23R* corrected for GAPDH and normalized to HCs on PBMCs. PMA/ionomycin = phorbol 12-myristate 13-acetate/ionomycin (4 h, 25 ng/mL + 1 µg/mL). Data pooled from 6 independent experiments, comparison by unpaired Student *T*-test. **b** IL23R surface (N-terminal antibody) staining on CD4^+^ T cells from HCs (*n* = 9, 2 shown) and patient (*n* = 1, 3 biological replicates, 1 shown). Fluorescence minus one (FMO) IL23R was used for proper gating. Percentage of IL23R^+^ cells from CD4^+^ and CD8.^+^ live cells, data pooled from 2 independent experiments. Mann–Whitney *U* test was used for comparison. **c** Confocal images of transfected (GFP) HeLa cells with WT, R381Q or R381X IL23R (C-terminally 3xHA tagged IL23R IRES GFP constructs), representative of 2 independent experiments. Counterstaining with DAPI. Scale bar is 5 μm. **d** IL23R surface (N-terminal antibody) staining on transfected HeLa cells with WT, R381Q or R381X IL23R (C-terminally 3xHA tagged IL23R IRES GFP constructs), representative of 2 independent experiments. **e** STAT3 phosphorylation in HeLa cells untransfected or cotransfected with IL12RB1, empty vector GFP (EV), IL23R WT, IL23R R381Q or IL23R R381X plasmids, unstimulated or stimulated with IL23 (100 ng/mL) or IFN-α (10 ng/mL) for 1 h, representative blot of 3 independent experiments. * indicates the STAT-3 band under αHA

*IL23R* undergoes extensive alternative splicing, with up to 24 isoforms produced, of which seven are not expressed due to nonsense-mediated decay and five of which are translated to produce a soluble form of IL23R (sIL23R) lacking an intracellular domain [10]. The remaining isoforms are expressed as membrane-bound receptors with varied extracellular domains. All transcripts encoding the putative isoforms contain exon 1, 2, and 3 and therefore we designed our primers to detect this region, measuring the total *IL23R* transcript level in PBMCs (Fig. 2a). As baseline expression of total *IL23R* is low, phorbol myristate acetate (PMA) and ionomycin were used to upregulate its transcription. Compared to healthy controls, the patient had tenfold lower *IL23R* mRNA levels both unstimulated and after PMA/ionomycin stimulation. Similar results were achieved using a primer–probe set covering the 3’ region, indicative of nonsense-mediated decay (FigS1a). Overexpression of a truncated IL23R (F380-HA) could not be detected (FigS1b) and flow cytometric detection of membrane-bound IL23R was severely decreased compared to healthy controls, although not completely absent in PBMCs or EBV-B cells of the patient (Fig. 2b, FigS1c-d). Thus, R381X results in near absent expression of membrane bound IL23R, primarily via nonsense-mediated decay.

### R381X Is a Complete Loss of Function Variant When Expressed in HeLa Cells

As IL-23 signals through the IL23R via JAK2 to induce STAT-3 phosphorylation, we first tested whether this response was blunted by the R381X mutation in a HeLa cells. Since IL23R functions as a heterodimeric receptor, we co-expressed both IL23R (WT, R381Q, or R381X) and IL12RB1 to assess the STAT3 phosphorylation response in HeLa cells. The presence of WT and R381Q IL23R on the membrane after transfection was confirmed by confocal microscopy (Fig. 2c) and flow cytometry (Fig. 2d), where it colocalized with IL12RB1 (FigS1e). As expected, R381X IL23R was neither expressed on the membrane nor in the cytosol of HeLa cells (Fig. 2c–d). After stimulation of HeLa cells with IL-23, we observed a strong STAT3 phosphorylation in WT cotransfected HeLa cells, while in the case of a cotransfection with IL23R R381Q or R381X, STAT3 phosphorylation was only moderately increased or unchanged, respectively, compared to untransfected or IL12RB1 with empty vector cotransfected cells (Fig. 2e). These findings indicate that the IL23R R381X is a complete LOF variant.

### Patient EBV-B Cells and PBMCs Have Impaired STAT3 Phosphorylation in Response to IL-23, Which Can Be Restored by Transduction of WT IL23R

**Fig. 3:**
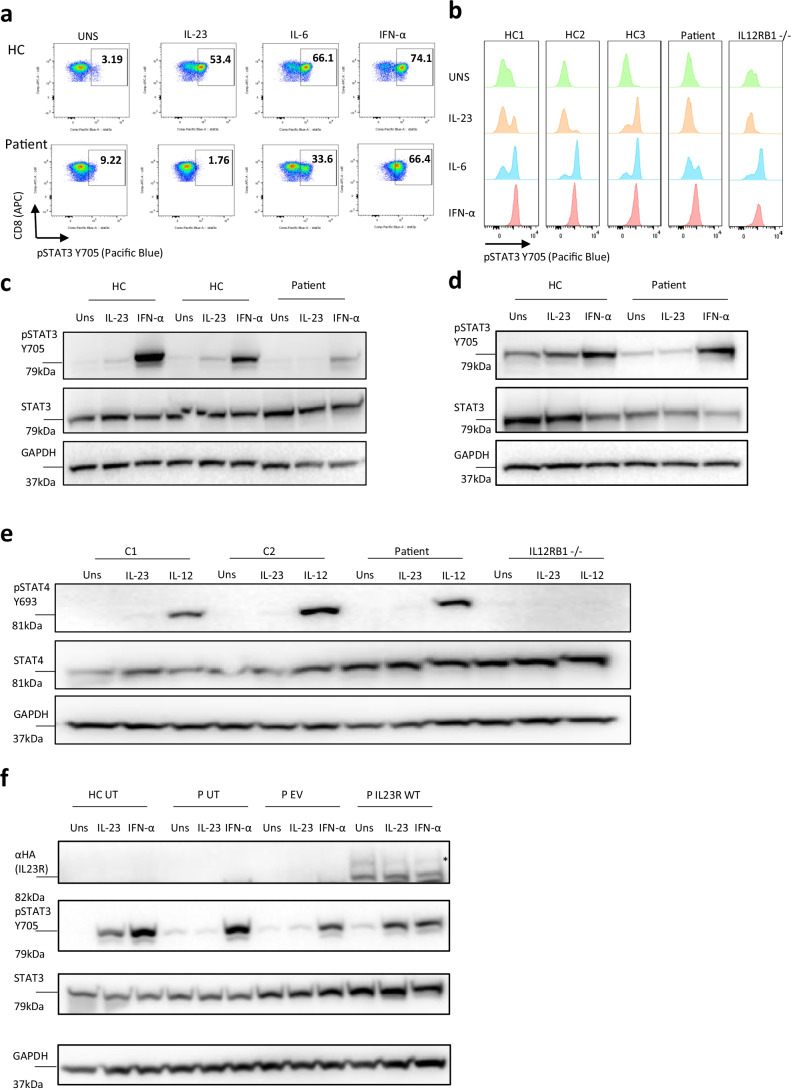
Impaired STAT3 but preserved STAT 4 phosphorylation responses in patient EBV-B cells and PBMCs. **a** FACS plot of 1 HC and patient for pSTAT3 on CD8 + T cells after IL-23 (100 ng/mL), IL-6 (10 ng/mL) or IFN-α (10 ng/mL) stimulation for 60 min. FMO pSTAT3 was used for proper gating. Representative of 2 independent experiments **b** pSTAT3 histograms from 3 HCs, patient and an IL12RB1 -/- patient, representative of 2 independent experiments **c** Western blot on EBV-B cells of 2 HC and patient for pSTAT3, stimulation as indicated in Fig. 3a. Representative of 3 independent experiments. **d** Western blot on PBMCs of 1 HC and patient for pSTAT3, stimulation as indicated in Fig. 3a. Representative of 3 independent experiments. **e** Western blot showing pSTAT4 after IL-23 (100 ng/mL) and IL-12 (40 ng/mL) stimulation of anti-CD3/CD28 pre-activated PBMCs from 2 HCs, patient and an IL12RB1 -/- patient. Data from 1 experiment. **f** pSTAT3 after lentiviral vector transduction of patient cells using stimulation as indicated in Fig. 3a, representative of n = 2 independent experiments. *N-glycosylated IL23R, UT = untransduced. EV = empty vector

**Fig. 4:**
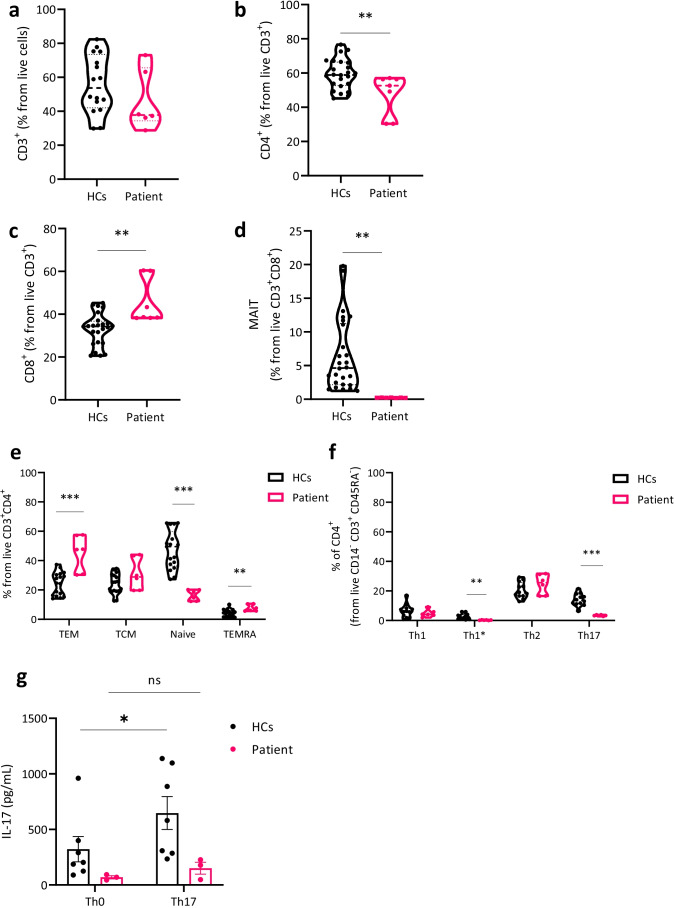
Reduced Th17, Th1*, and MAIT cells are characteristic for IL23R deficiency. **a** CD3^+^ T cells, **b** CD4^+^ T cells, **c** CD8^+^ T cells, **f** mucosal-associated invariant T (MAIT) cells (CD8^+^CD161^+^Vα7.2^+^), **e** proportion of T effector memory (TEM), T central memory (TCM), Naïve T cells and TEM re-expressing CD45RA (TEMRA) cells from CD3^+^CD4^+^ T cells. **f** proportion of Th1 (CCR4^−^CXCR3^+^RORγT^−^), Th1* (CCR4^−^CXCR3^+^RORγT^+^), Th2 (CCR4^+^CXCR3^−^RORγT^−^), and Th17 (CCR4^+^CXCR3^−^RORγT^+^) cells from live CD14^−^CD3^+^CD4^+^CD45RA^−^ cells. Pooled data from 3 independent experiments. Unpaired Student *T* test was used for comparison between groups. **g** IL-17 secretion after 7 days in vitro differentiation of naïve CD4.^+^ T cells (Th0) to Th17 cells. Pooled data from 3 independent experiments. Paired Student *T*-test was used for comparison within groups

Next, we evaluated IL-23 signaling in patient PBMCs and EBV-B cells using flow cytometry (Fig. 3a–b, FigS2) and Western blot (Fig. 3c–d, FigS3). In contrast to stimulation of patient PBMCs with IL-6 and IFN-α, which led to increased STAT3 phosphorylation, no increase was observed using IL-23, comparable to results seen in PBMCs of an IL12RB1-deficient patient (Fig. 3a–d). The IL-12 signaling pathway was still intact as a normal STAT4 phosphorylation response to IL-12 was observed in patient PBMCs (Fig. 3e, FigS4). To evaluate if IL-23 signaling could be restored in the patient, we introduced WT IL23R by lentiviral transduction in patient EBV-B cells (Fig. 3f). The introduction of WT IL23R, but not empty vector, in patient EBV-B cells restored STAT-3 phosphorylation upon IL-23 stimulation. These results provide a causal link between the observed defect in STAT-3 phosphorylation downstream of IL-23 and the IL23R R381X variant.

### IL23R Deficiency Is Characterized by Decreased Circulating Th17, MAIT, Th1* Cells, and Impaired Th17 Differentiation

**Fig. 5:**
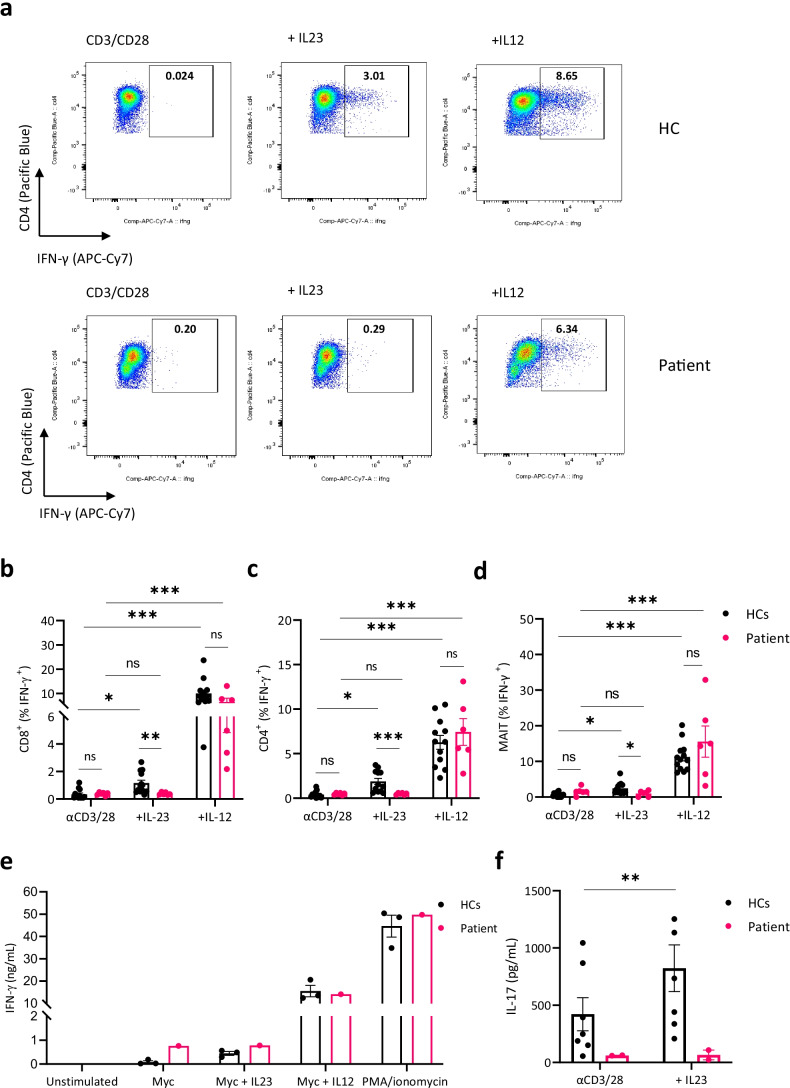
Impaired IFN-γ and IL-17 production in response to IL-23 stimulation. **a** FACS plots of IFN-γ secretion by CD4^+^ T cells in 1 HC and patient, pre-activated PBMCs with anti-CD3/CD28 with or without addition of IL-23 (100 ng/mL), IL-6 (10 ng/mL), or IL-12 (40 ng/mL) for 7 days. FMO IFN-γ was used for proper gating. Representative of 3 independent experiments **b–d** % of IFN-γ secreting CD8^+^, CD4.^+^, and MAIT cells, pooled from 3 independent experiments. To assess differences between conditions within HCs or patient, a Friedman test (*p* < 0.0001) with post hoc Dunn’s multiple comparison test was used. To compare HC and patient groups, a Mann–Whitney *U* test was used for each condition. **e** IFN-γ measured from supernatant of PBMCs in co-culture with *Mycobacterium abscessus* ATCC19977 for 3 days with or without IL-23 or IL-12. PMA ionomycin was used as a positive control condition. Data from 1 experiment. **f** IL-17 secreted by stimulated PBMCs with CD3/CD28 with or without addition of IL-23 (100 ng/mL), data from 2 experiments. To assess response in HCs, a paired Student *T*-test was used

To determine the impact of this signaling loss on the patient’s immune system, we first compared the frequencies of leukocyte subsets in healthy controls and patient. The frequencies of B, NK, total T cells, and regulatory T cells were normal in the patient (TableS1). Relative to healthy control frequencies, CD4^+^ T cells were decreased and CD8^+^ T cells increased, but absolute numbers remained within normal range (Fig. 4a–c, TableS1). The frequency of mucosal-associated invariant T (MAIT) cells (defined as CD8^+^Vα7.2 + CD161 + cells) was also severely decreased in the patient (Fig. 4d, FigS5). Further characterization of the CD4^+^ T cell compartment (Fig. 4e, FigS5) revealed a higher proportion of effector memory T cells (CD3^+^CD4^+^CD45RA^−^CCR7^−^) and T effector memory re-expressing CD45RA (TEMRA) cells (CD3^+^CD4^+^CD45RA^+^CCR7^−^), while naïve T cells (CD3^+^CD4^+^CD45RA^+^CCR7^+^) were decreased. In the CD4^+^ memory compartment (CD3^+^CD45RA^−^), the number of Th17 cells (CD3^+^CD45RA^−^CCR4^+^CXCR3^−^RORγT^+^) and Th1* (CD3^+^CD45RA^−^CCR4^−^CXCR3^+^RORγT^+^) were significantly decreased in the patient (Fig. 4f, FigS6-7). Given the low proportion of Th17 cells in the patient, we investigated the effect of IL-23 signaling on the generation of Th17 cells from naïve CD4^+^ T cells (CD45RA^+^) upon stimulation with polarizing cytokines in vitro. IL-17 secretion, a surrogate marker for Th17 differentiation, was low at baseline in the patient and did not increase in a Th17 polarizing condition in contrast to the healthy controls (Fig. 4g). These results are consistent with the known functions of IL-23 in lymphocyte biology, supporting a loss of IL-23 signaling in the patient.

### IFN-γ and IL-17 Secretion by CD8^+^, CD4.^+^ T, and MAIT Cells in Response to IL-23 Is Blunted in the Patient

Based on previous observations, identifying IL-23 as a stimulator of IFN-γ especially in alpha–beta T cell subsets and MAIT cells [7], we tested whether the patient alpha–beta T cell subsets and MAIT cells had impaired IFN-γ secretion after IL-23 stimulation. Therefore, we incubated PBMCs in the presence of anti-CD3/anti-CD28 and added conditions with or without IL-23, IL-12, or IL-6. Addition of IL-12 resulted in a significant increase of IFN-γ production compared with the unstimulated condition in CD8^+^, CD4^+^, and MAIT cells from both healthy controls and the patient. However, we observed that addition of IL-23 was unable to induce IFN-γ production in the patient in contrast to the healthy controls (Fig. 5a–d, Fig S8a-b). Similarly, this defect of IFN-γ secretion in response to IL-23 was also seen in patient NK and iNKT cells while IFN-γ responses to IL-12 remained normal (FigS9a-c). To characterize the consequences of this functional defect in the presence of a mycobacterial infection, we co-cultured healthy control and patient PBMCs with non-tuberculous mycobacteria to assess the mycobacteria specific IFN-γ. In the absence of available strains identified in our patient, the *Mycobacterium abscessus* strain (ATCC19977) was used. The addition of IL-23 in the patient did not result in an augmented IFN-γ response compared to *Mycobacterium abscessus* alone, in contrast to healthy controls where a mild increase in IFN-γ production was seen (Fig. 5e, FigS10). This defect was specific to IL-23, since addition of IL-12 to mycobacteria or PMA/ionomycin as single stimulant resulted in a comparable increase of IFN-γ production. Next, we measured IL-17 production in the supernatant of stimulated PBMCs with anti-CD3/anti-CD28 with or without addition of IL-23. IL-17 secretion was low in both conditions and did not increase upon IL-23 stimulation, whereas healthy controls had higher baseline levels but increased upon IL-23 costimulation (Fig. 5f).

## Discussion

We report on a novel, homozygous *IL23R* (p.R381X) mutation as a cause of MSMD. IL23R is a heterodimeric receptor consisting of an IL12RB1 and IL23R subunit. After binding to IL-23, it signals downstream through TYK2 (attached to IL12RB1) and JAK2 (attached to IL23Rα) to induce phosphorylation of STAT3. The role of IL-23 signaling is best studied in the context of inflammatory disease. A well-described example is inflammatory bowel disease, where both mouse models, human observational studies and population genetic studies underscore the importance of IL-23 [11]. The connection of IL-23 signaling to mycobacterial disease is based on the observation that *IL23a*^*−/−*^ mice have impaired long-term control of pulmonary *Mycobacterium tuberculosis* infection [12], the fact that exogenous IL-23 can complement IL-12 deficiency for restoring mycobacterial immunity in mice [13], the association of a partial LOF variant c.1142G > A (R381Q) with active pulmonary tuberculosis [8] and the report of a kindred with MSMD harboring a homozygous complete LOF mutation in *IL23R* [7]. In addition, a second patient with MSMD and a splice homozygous splice site mutation, c.367 + 1G > A, at the exon 3 of *IL23R* is reported but the effect of this variant on IL23R expression and signaling was not reported [14].

In contrast to the first reported kindred by Martínez-Barricarte et al. where both patients had a homozygous missense mutation (p.C115Y) in the extracellular domain resulting in impaired IL-23 signaling, our patient has an adult-onset phenotype and a stop mutation (p.R381X) located in the intracellular domain before the JAK2 binding site. The mutation resides in a well-conserved residue and was predicted to be LOF using in silico prediction tools. Functional testing revealed that our patient had severely decreased total *IL23R* mRNA levels and membrane-bound IL23R. The functional impact of this mutation was assessed by determining STAT3 phosphorylation following IL-23 stimulation in primary cells and in HeLa cells transfected with WT IL23R or the partial LOF variant R381Q [15, 16]. The R381X mutation in our patient has a complete LOF effect in both overexpression and primary cells, which can be restored by lentiviral transduction of WT IL23R. The immunophenotype of the IL23R-deficient patient was characterized by a low number of MAIT cells, resembling the previously reported IL23R patient but also STAT3, IL12RB1 andTBX21 patients [6, 17]. Furthermore, Th17 and Th1* cells were significantly reduced, differing from the previously reported patient where only a mild reduction was observed [7]. In addition, the relative proportion of CD4^+^ and CD8^+^ T cells in our patient differed from healthy controls in contrast to the previously reported IL23R deficient patient [7]. The effect of a homozygous stop mutation on Th17 cell development is consistent with previous data reporting reduced circulating Th17 cells in individuals carrying a heterozygous R381Q mutation [16]. Moreover, the observation of reduced Th17 cells is supported by studies demonstrating a central function for IL-23 in TH17 differentiation in mice [18]. In addition, we showed that the differentiation of naïve T cells to Th17 was impaired, confirming previous results [7]. Remarkably, our patient did not have any susceptibility to fungal infections. This supports the hypothesis that—at least in humans—the contribution of IL-23- to IL-17-mediated anti-fungal immunity might be redundant [7].

Finally, we assessed IFN-γ secretion by alpha–beta T cell subsets (CD4^+^, CD8^+^) and MAIT cells upon IL-23 stimulation. IL-23 is a less potent IFN-γ inducer in T cells compared to IL-12 as described by Martínez-Barricarte et al., characterizing IL-12- and IL-23-specific effects in different immune subsets [7]. We observed a mild, but significant increase of IFN-γ production by alpha–beta T cell subsets and MAIT cells in healthy controls when stimulated with IL-23, while this was absent in the patient. IL-12 stimulation remained intact, confirming a selective IL-23 signaling defect. Furthermore, in the context of a non-tuberculous mycobacterial infection in vitro, the addition of IL-23 did not increase IFN-γ in the patient, in contrast to healthy controls. In addition, IL-17 secretion by stimulated PBMCs in the patient was low and did not increase upon IL-23 costimulation.

In conclusion, we identified a patient with MSMD and a novel stop mutation (p.R381X) located in the intracellular domain of IL23R. This report illustrates a mechanism of abolished membrane-bound IL23R expression. Functionally, this negatively affects the development of Th17 and MAIT cells and impacts IL-23-stimulated IL-17 and IFN-γ production by alpha–beta T cell subsets and MAIT cells. Our findings together with the report of a previously validated IL23R patient with MSMD and existing data on mycobacterial susceptibility of *IL23a*^*−/−*^ mice link the genotype and phenotype in our patient. The absence of fungal infections in this adult patient and the previously reported pediatric onset patients are consistent with the hypothesis that the contribution of IL-23- to IL-17-mediated anti-fungal immunity might be redundant, despite the presence of decreased circulating Th17 cells and reduced IL-17 secretion in response to IL-23.

## Supplementary Information

Below is the link to the electronic supplementary material.

Supplementary file1 (PDF 1902 KB)

## Data Availability

The authors confirm that the data supporting the findings of this study are available within the article and/or its supplementary material.
