# Supplementary material for: A Novel Homozygous Stop Mutation in IL23R Causes Mendelian Susceptibility to Mycobacterial Disease
Source: J Clin Immunol. 2022 Jul 13;42(8):1638–52. doi: 10.1007/s10875-022-01320-7 (PMC9700586; doi:10.1007/s10875-022-01320-7)
Supplement: Supplementary file 1 — Supplementary file1 (PDF 1902 KB) [file 10875_2022_1320_MOESM1_ESM.pdf]

Supplementary data

Detailed clinical case description

A 48-year old male patient from Turkish descent, born to non-consanguineous parents, presented at the age of 41 with complaints of progressive weight loss, intermittent fever, night sweats, general weakness and exertional dyspnea. He was an active smoker and had a medical history of chronic obstructive pulmonary disease (COPD). Laboratory evaluation (**Table S1**) showed a marked inflammation and microcytic anemia. Microbial cultures, syphilis, toxoplasmosis and viral serology (HIV, HBV, HCV, CMV, EBV) were negative. Positron emission tomography (PET)- computed tomography (CT) scan revealed the presence of hepatosplenomegaly accompanied by diffuse hypermetabolic intrathoracic and abdominal adenopathies. In addition, the bone marrow appeared hyperreactive. A biopsy of an intrathoracic lymph node was acquired by endobronchial endoscopy and *Mycobacterium avium* complex (MAC) was cultured. A gastroscopy with gastric and duodenal biopsies also revealed acid-fast bacilli, with negative culture but positive PCR identifying *Mycobacterium tilburgii*. IGRA (Quantiferon-TB) and tuberculin lymphocyte stimulation test were both negative. A treatment regimen of tuberculostatics (ethambutol, rifampicin and clarithromycin) was initiated. However, symptoms of weight loss, fatigue and systemic inflammation persisted despite adequate compliance to treatment and no evidence of mycobacterial resistance. On second admission, 7 months after treatment initiation, laboratory evaluation showed a persistent C-reactive protein (CRP) of 110 mg/dl and microcytic anemia (Hb 7.9 g/dl, ref 14-18, MCV 74 f), acid fast bacilli and histiocytic proliferation were identified on bone marrow, gastric/duodenal biopsy and sputum, confirming a disseminated mycobacterial infection. Cultures and PCR remained negative. The tuberculostatic treatment was continued. After 9 months of treatment initiation a PET CT scan still showed hypermetabolic enlarged intrathoracic and abdominal adenopathies. A supraclavicular lymph node excision biopsy was performed, revealing histiocytic proliferation but no evidence of acid-fast bacilli on staining. Cultures and PCR were negative. Tuberculostatic treatment was stopped after a total duration of 10 months, given the absence of any significant amelioration and no detectable mycobacterial infection. Thereafter, the patient clinically improved and a PET CT scan, 7 months after treatment cessation, was normal. However, systemic inflammation (CRP) remained increased for more than 3 years (CRP ≥10 mg/dL) and symptoms of fatigue persisted. At the point of experimental assessment, the

patient was in a good condition, without any evidence of mycobacterial infection and no residual inflammation. He was last admitted at the age of 47 for a COPD infectious exacerbation (*Haemophilus influenzae*) with hypercapnic respiratory failure requiring non-invasive ventilation. Immunological evaluation throughout his disease course, including immunoglobulin levels, T/B/NK cell counts, lymphocyte proliferation (on PHA, CD3, IL2, CD3+IL2, tetanus toxoid), neutrophilic oxidative burst (DHR), TLR functionality, IFN-γ auto-antibodies and STAT4 phosphorylation upon IL-12 stimulation appeared normal. Targeted sequence panels for primary immunodeficiency (PID) genes including *GATA2*, *STAT1* was negative. Eventually whole-exome sequencing was performed showing a homozygous stop mutation (c.1141C>T, p.R381X) in *IL23R*. This mutation was not reported in homozygous state in a healthy population database (gnomAD V3, 1000 Genomes or ExAC) and predicted damaging based on CADD (37), SIFT (1). Segregation analysis revealed that he was the only member carrying this variant in homozygosity.

	**Reference**	**Last follow up**	**PET-CT scan (+17M)**	**Treatment cessation (+10M)**	**Second admission (+6M)**	**First admission – diagnosis and start treatment (T0)**
**CBC**						
Hemoglobin (g/dL)	14-18	16	**8.6**	**7.6**	**7.9**	**7.7**
Leukocyte (x10^9/L)	4-10	5.6	**11.65**	7.52	**10.37**	**5.9**
Thrombocytes (x10^9/L)	150-400	212	**499**	495	343	250
Neutrophils (x10^9/L)	2.5-7.8	3.3	**9.7**	5.8	**8.4**	
Eosinophils (x10^9/L)	≤0.4	0.2	0.0	0.1	0.0	
Lymphocytes(x10^9/L)	1.2-3.6	1.7	1.2	**1.1**	1.2	
Basophils (x10^9/L)	≤0.1	0.1	0.0	0.0	0.0	
C-reactive protein (mg/dL)	≤5	2.5	**152.9**	**119.6**	**110**	**80.7**
**Immunoglobulin**		ND	ND			ND
IgG (g/L)	7.51-15.6			**17.8**	**20.1**	
IgA (g/L)	0.82-4.53			2.71	2.93	

IgM (g/L)	0.46-3.04			**3.23**	**3.97**	
**Immune subsets + functionality**		ND	ND			ND
CD3 + T cells (x10^9/L)	0.798-2.823			1.296		
CD4 + T cells (x10^9/L)	0.455-1.885			0.720		
CD8 + T cells (x10^9/L)	0.219-1.124			0.479		
Regulatory T cells (CD25+ CD127 low, % of CD4)	5-12			6.8		
Naïve T cells (CD27+CD45RA+, % of CD3+)	/			21.3		
NKT cells (CD56+, % of CD3+)	≤20			11.8		
CD19+ B cells (x10^9/L)	0.082-0.476			0.113		
smBcells (CD27+IgM-IgD-, % of CD19+ B cells)	/			7.7		
CD3- CD56+ NK cells (x10^9/L)	0.066-0.746			0.149		
LTT IL-2	10^3 cpm				1.05	
LTT CD3	10^3 cpm				3.05	
LTT CD3+ IL2	10^3 cpm				0.79	
LTT CD3 + PMA	10^3 cpm				1.15	
LTT PMA+ionomycin	10^3 cpm				1.2	
Dihydrorhodamine oxidation (DHR)			Normal oxidative neutrophilic burst (75% of neutrophils)			
**Autoimmune serology**	ND	ND	ND	ND		
ANA					Neg	
ANCA						
**Microbial serology**		ND	ND	ND		
HIV Ab/Ag	Neg/Pos				Neg	
HBVsAg	Neg/Pos				Neg	
HBVsAb	Neg/Pos				Neg	

HCV Ab	Neg/Pos				Neg	
CMV IgM	Neg/Pos				Neg	
CMV IgG	Neg/Pos				Pos (>250 AU/mL)	
EBV IgM	Neg/Pos				Neg	
EBV EBNA	Neg/Pos				Pos	
Brucella IgM	Neg/Pos				Neg	
Brucella IgG	Neg/Pos				Neg	
Coxiella burnetii IgM	Neg/Pos				Neg	
Coxiella burnetii IgG	Neg/Pos				Neg	
**Mycobacterial culture and PCR**		ND				
Sputum					Neg (acid-fast staining pos, culture and PCR neg)	Neg (acid-fast staining pos, culture and PCR neg)
Bone marrow biopsy					Neg (acid-fast staining pos, culture and PCR neg)	
Lymph node biopsy				Neg (supraclavicular lymph node)		Pos (culture and PCR *Mycobacterium avium* complex)
Urine					Neg	
Blood			Neg	Neg	Neg	
Gastric/duodenal biopsy					Neg (acid-fast staining pos, PCR neg)	Pos (PCR *Mycobacterium tilburgii*)

Table S1: Laboratory data of the patient over time. Bold values exceed reference range values. LTT: lymphocyte transformation test. ND: not determined. HBV: hepatitis B virus.

HCV: hepatitis C virus. CMV: cytomegalovirus. EBV: Epstein Barr virus. ANA: anti-nuclear antibody. ANCA: anti neutrophilic cytoplasmic antibodies. Neg: negative. Pos: positive.

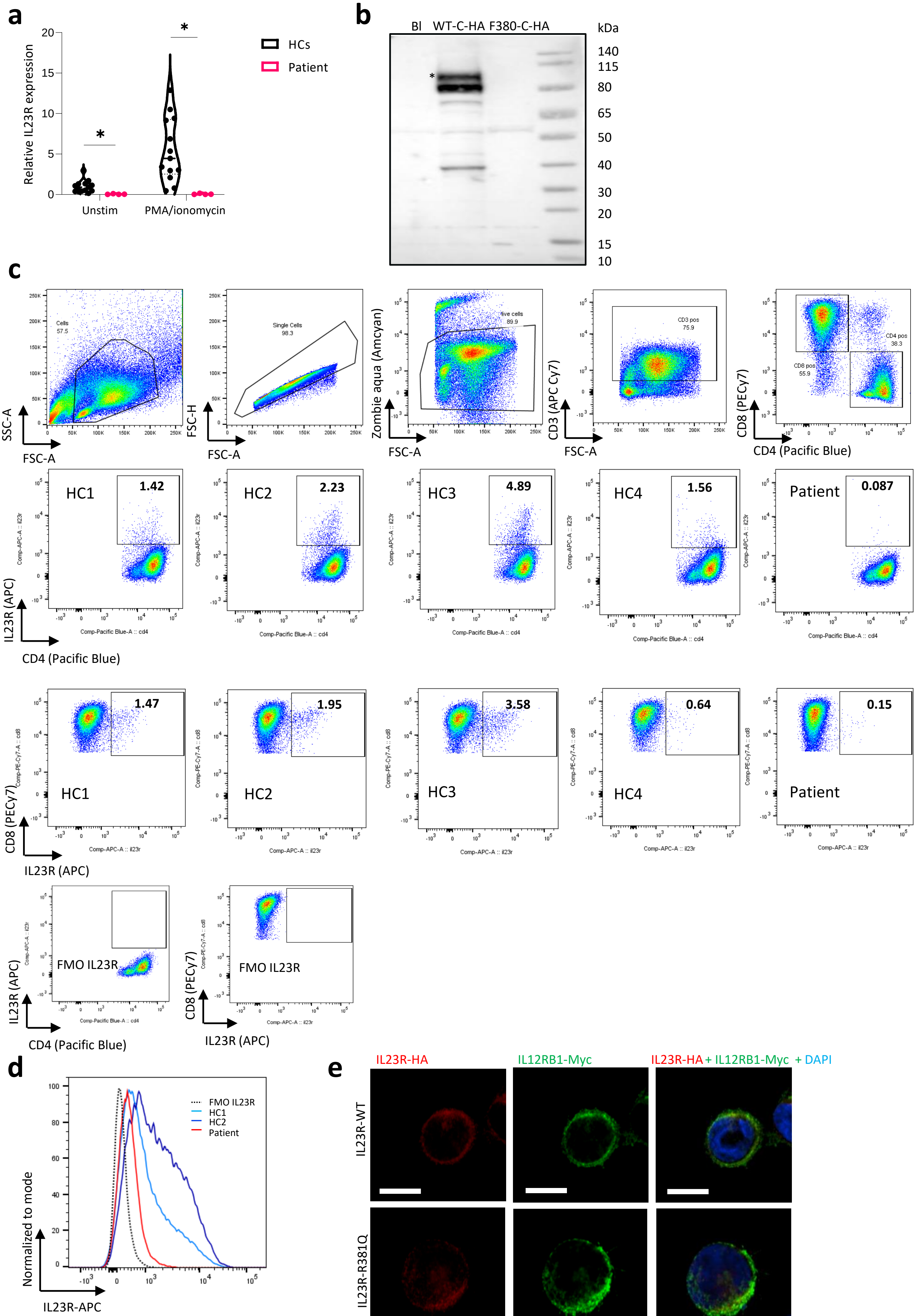
a
Relative IL23R expression
HCs
Patient
Unstim
PMA/ionomycin
b
Bl
WT-C-HA
F380-C-HA
kDa
140
115
80
65
50
40
30
20
15
10
c
SSC-A
FSC-A
FSC-H
Zombie aqua (Amcyan)
CD3 (APC Cy7)
CD8 (PECy7)
CD4 (Pacific Blue)
Cells
57.5
Single Cells
98.3
live cells
89.9
CD3 pos
75.9
CD4 pos
38.3
CD8 pos
55.9
HC1
1.42
HC2
2.23
HC3
4.89
HC4
1.56
Patient
0.087
IL23R (APC)
1.47
1.95
3.58
0.64
0.15
FMO IL23R
d
FMO IL23R
HC1
HC2
Patient
Normalized to mode
IL23R-APC
e
IL23R-HA
IL12RB1-Myc
IL23R-HA + IL12RB1-Myc + DAPI
IL23R-WT
IL23R-R381Q

FigS1

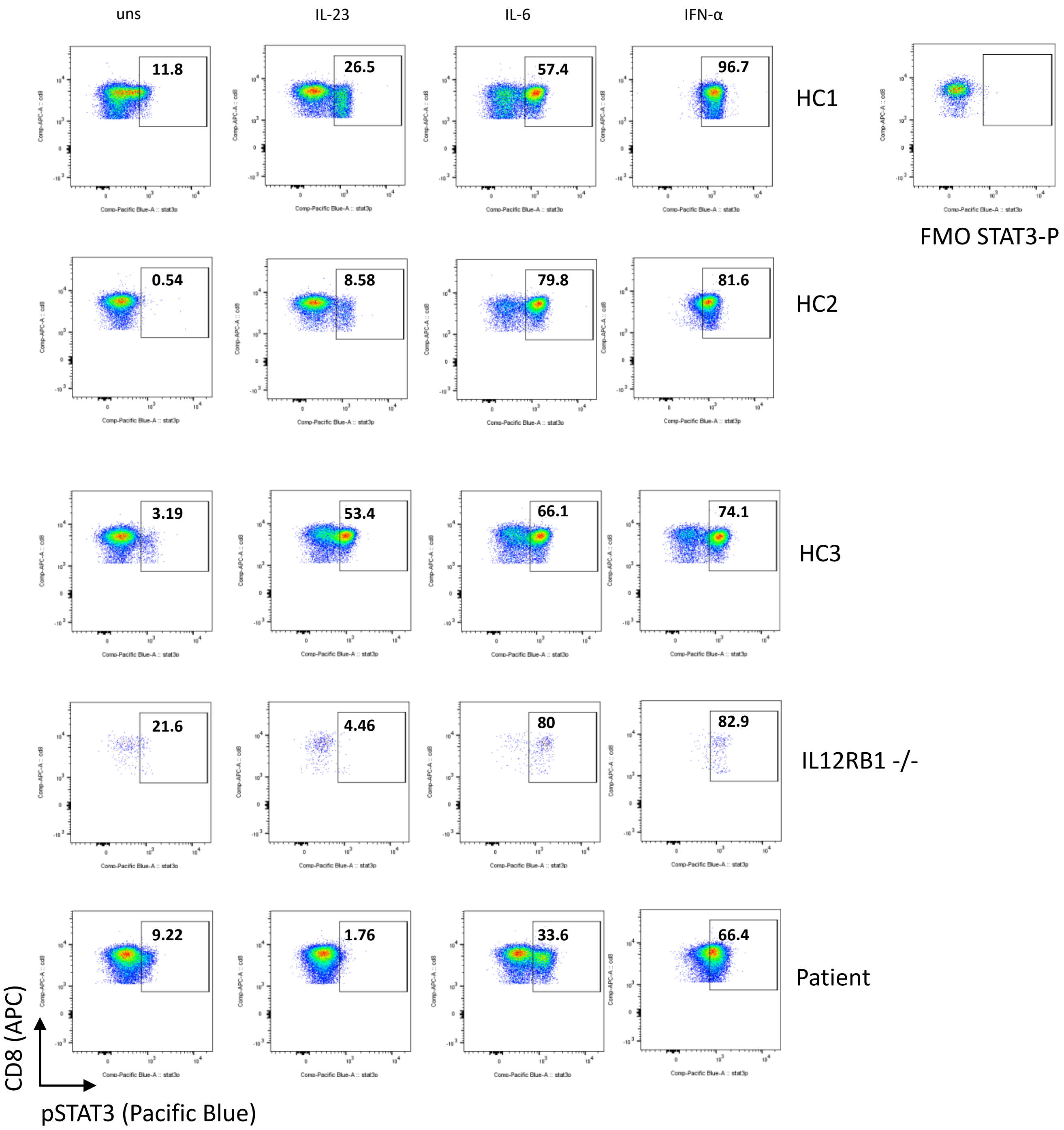
uns
IL-23
IL-6
IFN-α
11.8
26.5
57.4
96.7
HC1
FMO STAT3-P
0.54
8.58
79.8
81.6
HC2
3.19
53.4
66.1
74.1
HC3
21.6
4.46
80
82.9
IL12RB1 -/-
9.22
1.76
33.6
66.4
Patient
CD8 (APC)
pSTAT3 (Pacific Blue)

FigS2

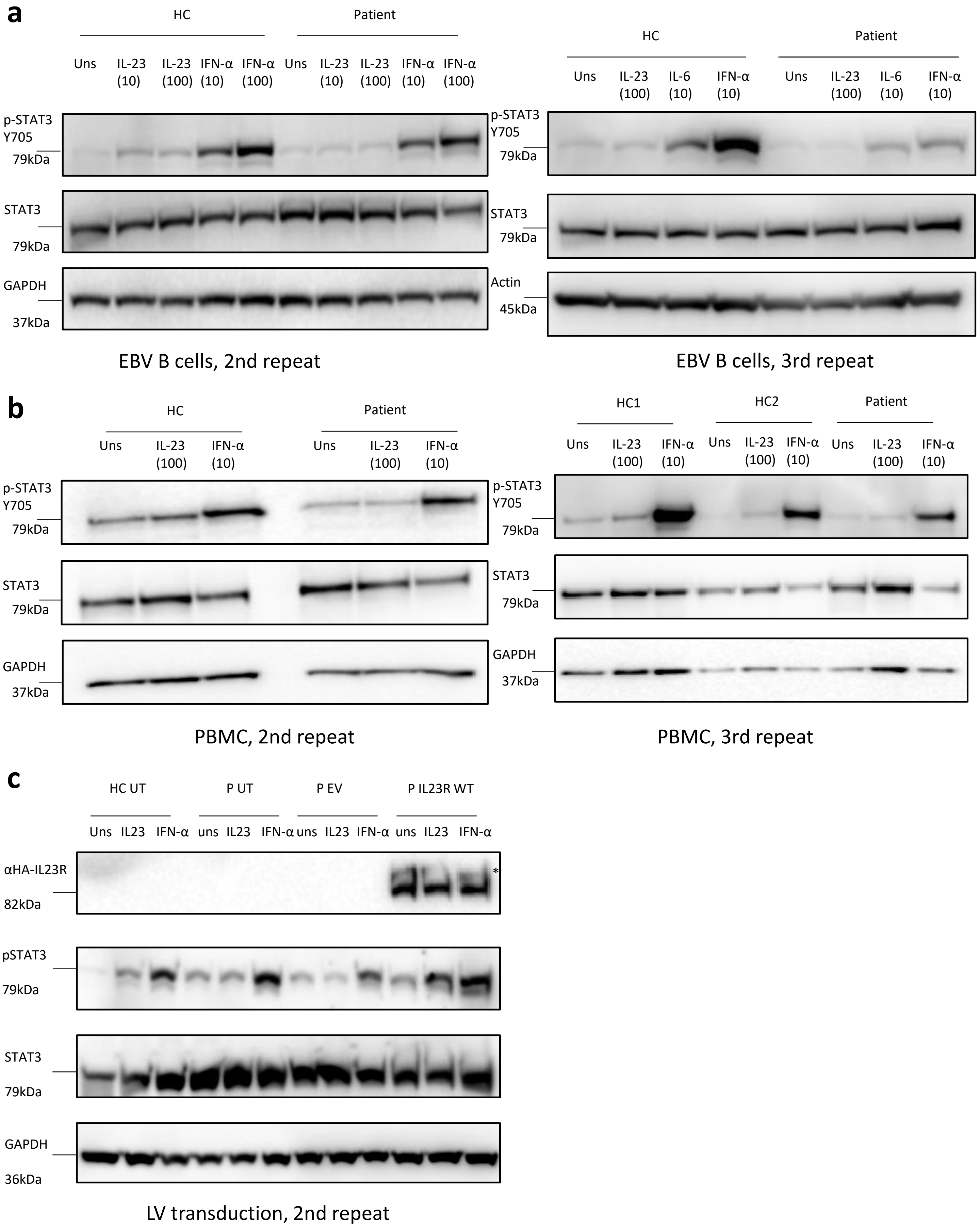
a
HC
Patient
Uns
IL-23 (10)
IL-23 (100)
IFN-α (10)
IFN-α (100)
IL-6 (10)
p-STAT3 Y705
79kDa
STAT3
GAPDH
37kDa
Actin
45kDa
EBV B cells, 2nd repeat
EBV B cells, 3rd repeat
b
HC1
HC2
PBMC, 2nd repeat
PBMC, 3rd repeat
c
HC UT
P UT
P EV
P IL23R WT
uns
IL23
IFN-α
αHA-IL23R
82kDa
*
pSTAT3
36kDa
LV transduction, 2nd repeat

FigS3

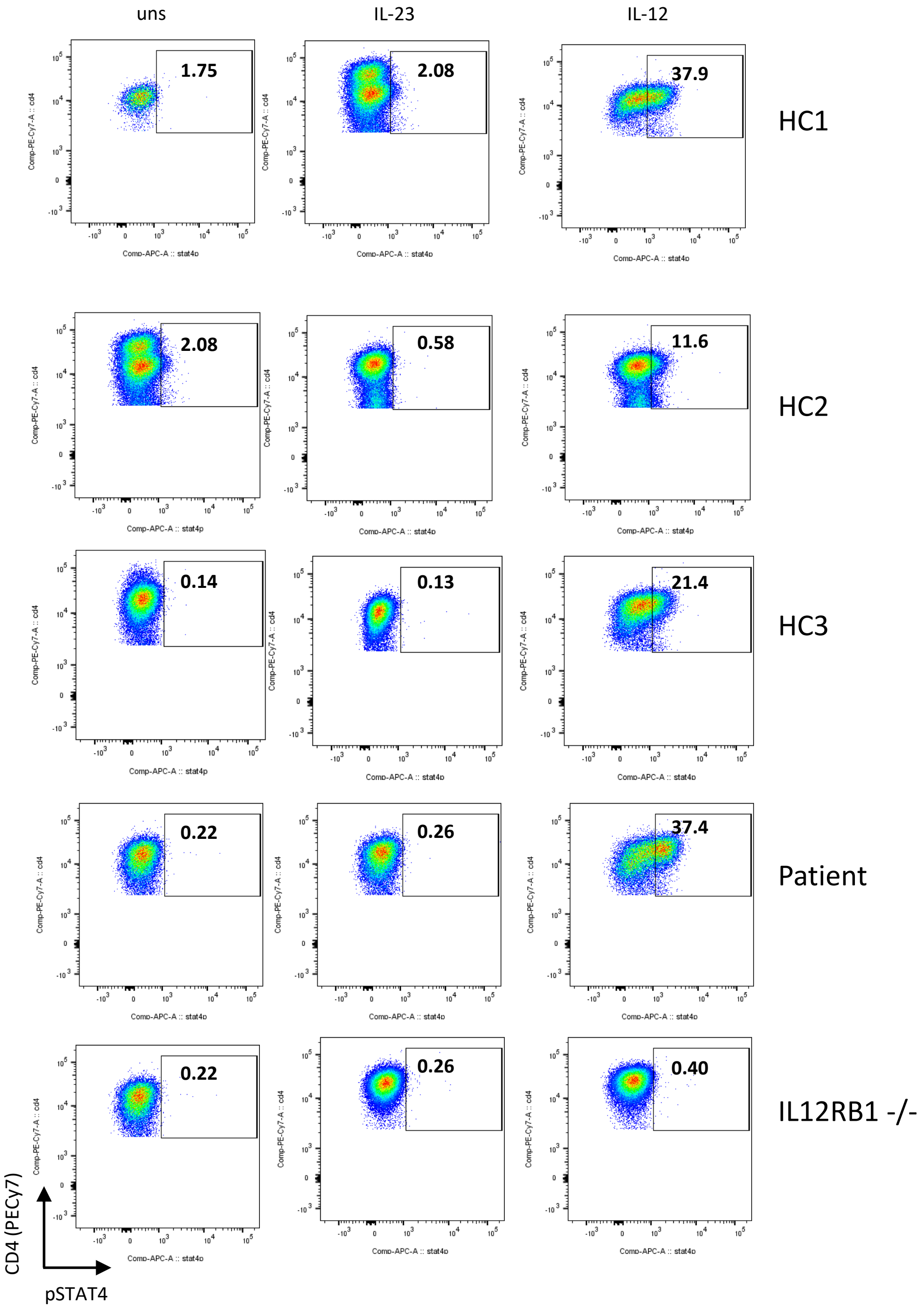
uns
IL-23
IL-12
1.75
2.08
37.9
HC1
2.08
0.58
11.6
HC2
0.14
0.13
21.4
HC3
0.22
0.26
37.4
Patient
0.22
0.26
0.40
IL12RB1 -/-
CD4 (PECy7)
pSTAT4
Comp-PE-Cy7-A :: cd4
Comp-APC-A :: stat4p

FigS4

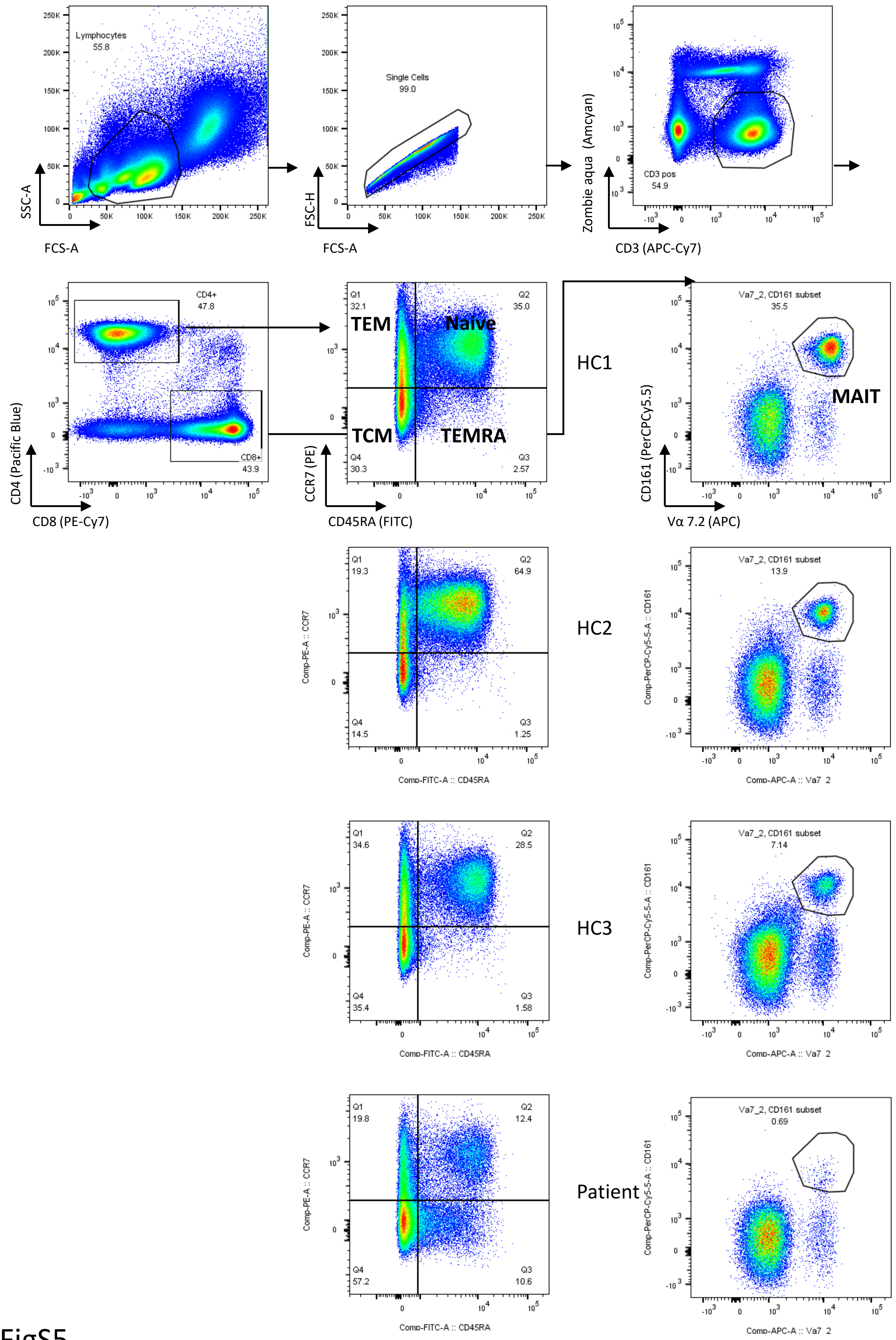
Lymphocytes
55.8
SSC-A
FCS-A
Single Cells
99.0
FSC-H
FCS-A
CD3 pos
54.9
Zombie aqua (Amcyan)
CD3 (APC-Cy7)
CD4+
47.8
CD8+
43.9
CD4 (Pacific Blue)
CD8 (PE-Cy7)
Q1
32.1
Q2
35.0
TEM
Naive
TCM
TEMRA
Q4
30.3
Q3
2.57
CCR7 (PE)
CD45RA (FITC)
HC1
Va7_2, CD161 subset
35.5
MAIT
CD161 (PerCPCy5.5)
Vα 7.2 (APC)
Q1
19.3
Q2
64.9
Q4
14.5
Q3
1.25
Comp-PE-A :: CCR7
Comp-FITC-A :: CD45RA
HC2
Va7_2, CD161 subset
13.9
Comp-PerCP-Cy5-5-A :: CD161
Comp-APC-A :: Va7_2
Q1
34.6
Q2
28.5
Q4
35.4
Q3
1.58
Comp-PE-A :: CCR7
Comp-FITC-A :: CD45RA
HC3
Va7_2, CD161 subset
7.14
Comp-PerCP-Cy5-5-A :: CD161
Comp-APC-A :: Va7_2
Q1
19.8
Q2
12.4
Q4
57.2
Q3
10.6
Comp-PE-A :: CCR7
Comp-FITC-A :: CD45RA
Patient
Va7_2, CD161 subset
0.69
Comp-PerCP-Cy5-5-A :: CD161
Comp-APC-A :: Va7_2

FigS5

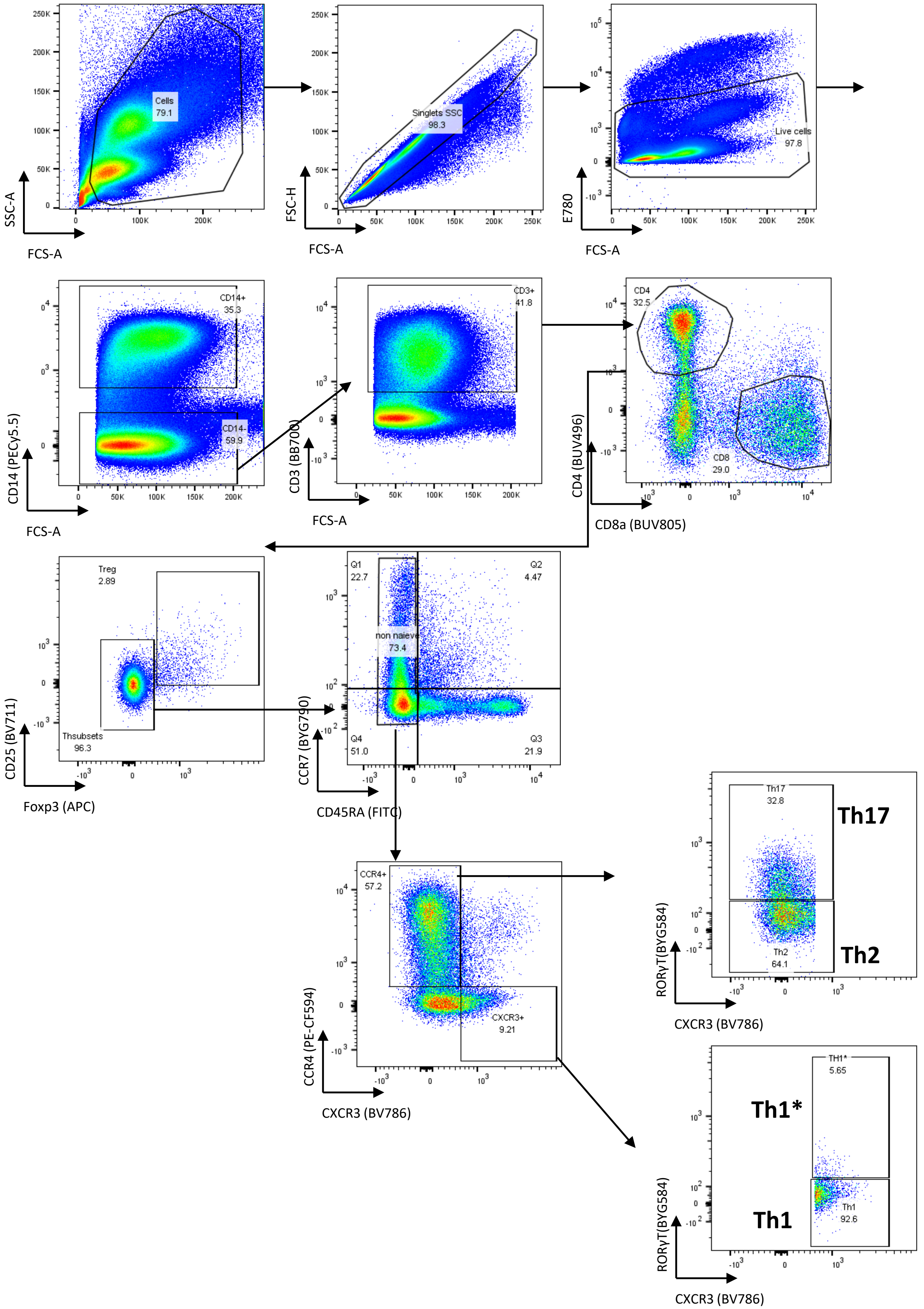

Cells
79.1
SSC-A
FCS-A
Singlets SSC
98.3
FSC-H
FCS-A
Live cells
97.8
E780
FCS-A
CD14+
35.3
CD14-
59.9
CD14 (PECy5.5)
FCS-A
CD3+
41.8
CD3 (BB700)
FCS-A
CD4
32.5
CD8
29.0
CD4 (BUV496)
CD8a (BUV805)
Treg
2.89
Thsubsets
96.3
CD25 (BV711)
Foxp3 (APC)
Q1
22.7
Q2
4.47
non naieve
73.4
Q4
51.0
Q3
21.9
CCR7 (BYG790)
CD45RA (FITC)
CCR4+
57.2
CXCR3+
9.21
CCR4 (PE-CF594)
CXCR3 (BV786)
Th17
32.8
Th17
Th2
64.1
Th2
RORγT(BYG584)
CXCR3 (BV786)
TH1*
5.65
Th1*
Th1
92.6
Th1
RORγT(BYG584)
CXCR3 (BV786)

FigS6

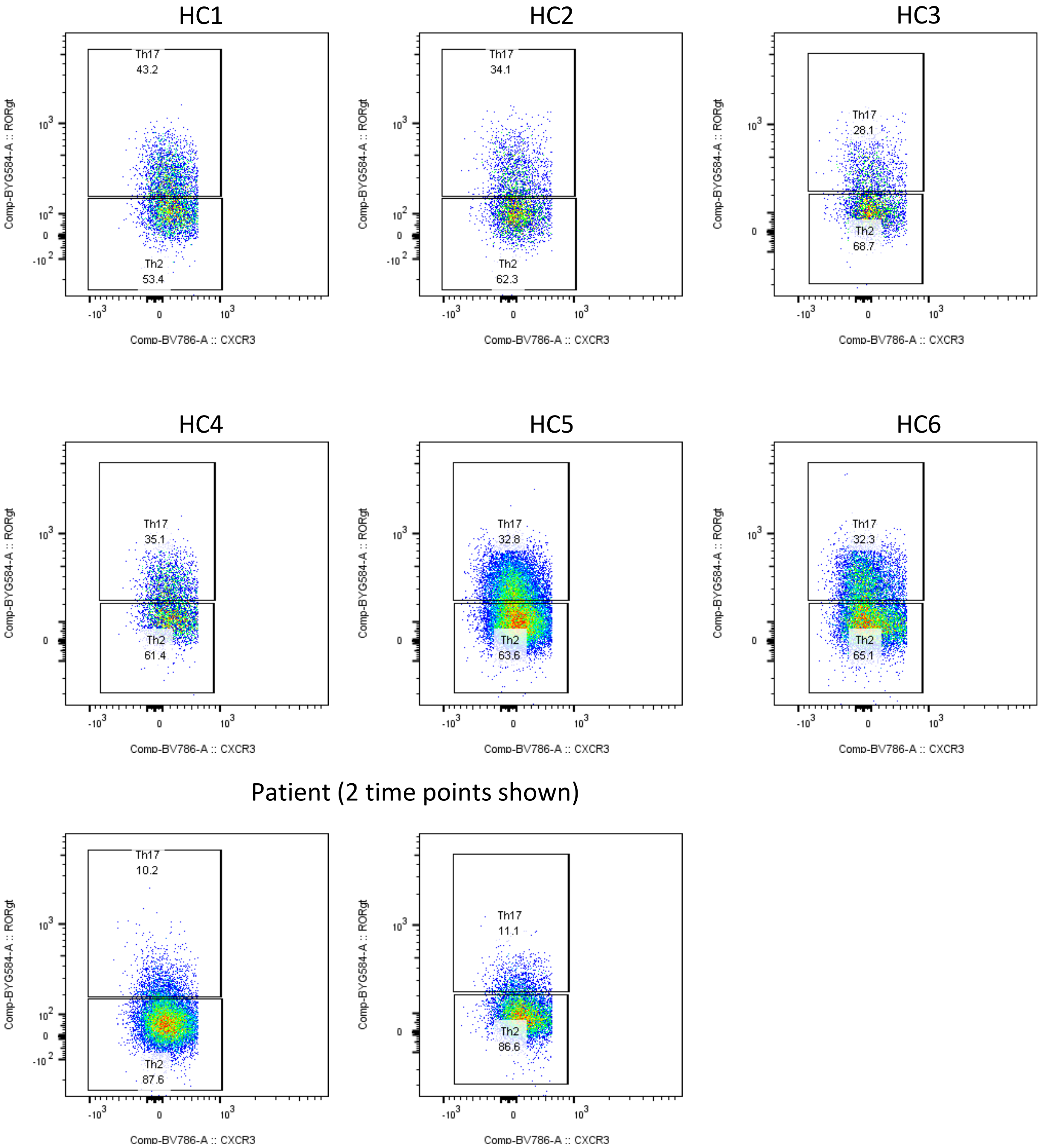
HC1
Th17
43.2
Th2
53.4
HC2
Th17
34.1
Th2
62.3
HC3
Th17
28.1
Th2
68.7
HC4
Th17
35.1
Th2
61.4
HC5
Th17
32.8
Th2
63.6
HC6
Th17
32.3
Th2
65.1
Patient (2 time points shown)
Th17
10.2
Th2
87.6
Th17
11.1
Th2
86.6
Comp-BYG584-A :: RORgt
Comp-BV786-A :: CXCR3

FigS7

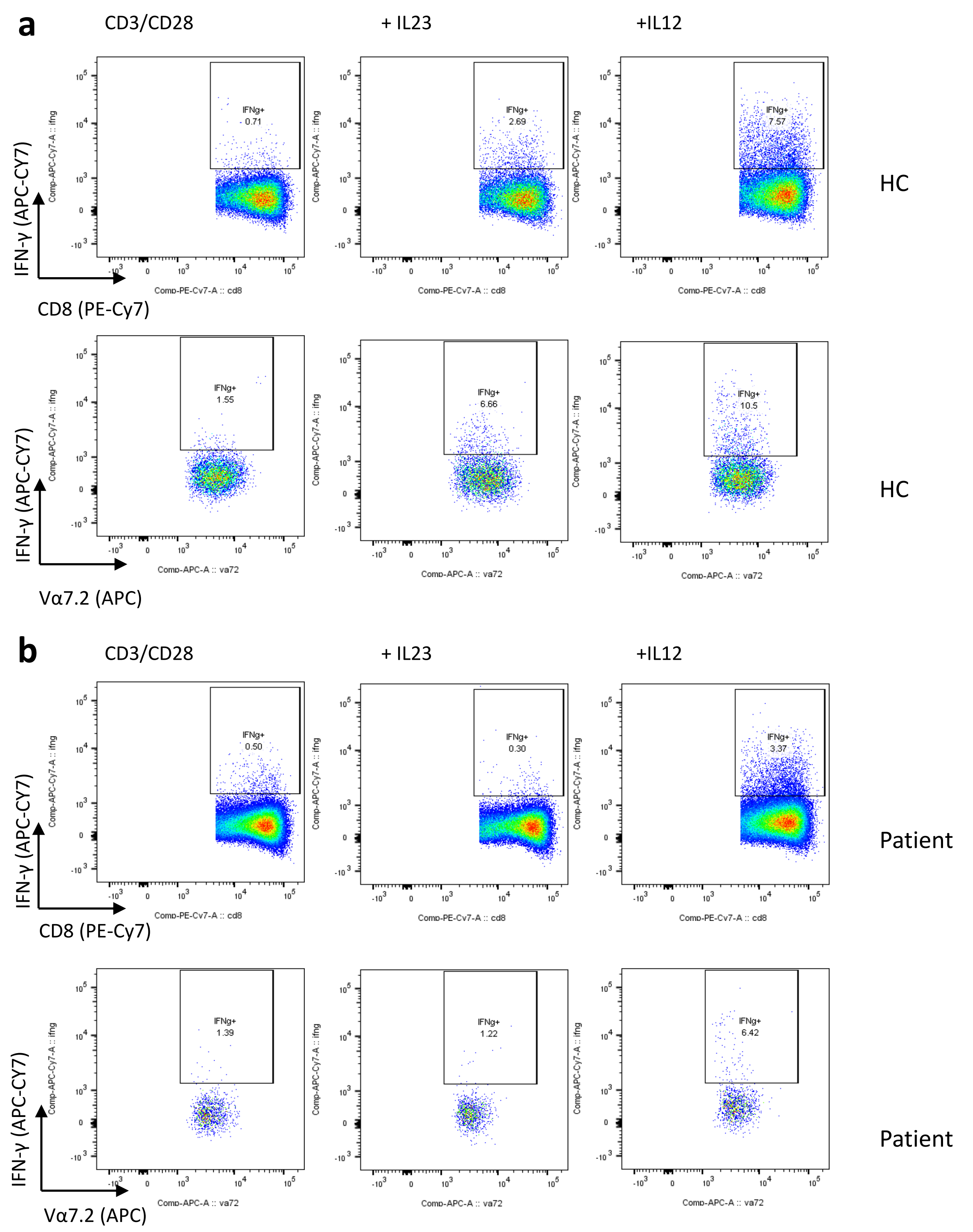
a
CD3/CD28
+ IL23
+IL12
HC
IFN-γ (APC-CY7)
CD8 (PE-Cy7)
Vα7.2 (APC)
IFNg+
0.71
2.69
7.57
1.55
6.66
10.5
Comp-APC-Cy7-A :: ifng
Comp-PE-Cy7-A :: cd8
Comp-APC-A :: va72
b
Patient
0.50
0.30
3.37
1.39
1.22
6.42

FigS8

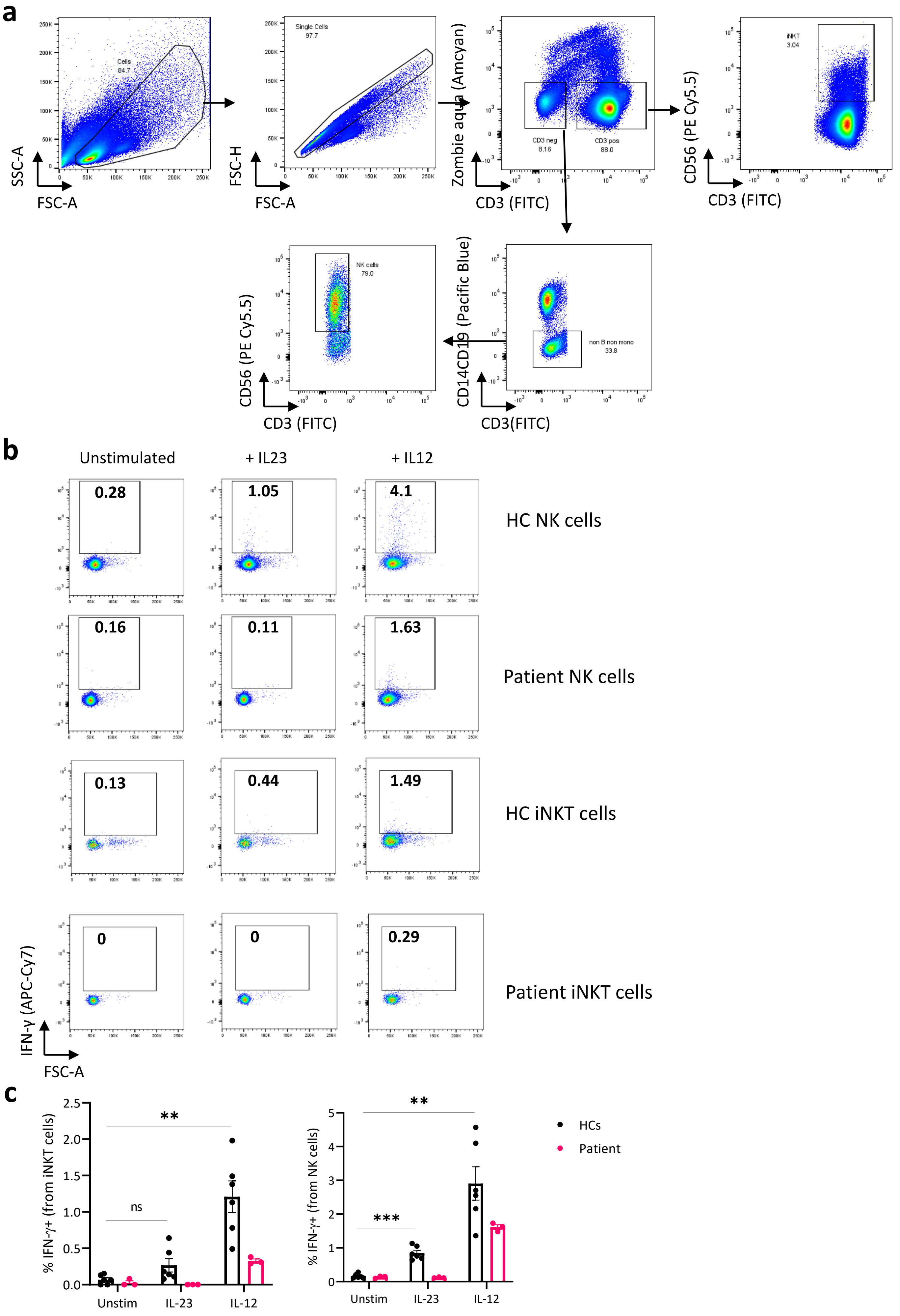
a
Cells
84.7
SSC-A
FSC-A
Single Cells
97.7
FSC-H
FSC-A
Zombie aqua (Amcyan)
CD3 neg
8.16
CD3 pos
88.0
CD3 (FITC)
iNKT
3.04
CD56 (PE Cy5.5)
CD3 (FITC)
NK cells
79.0
CD56 (PE Cy5.5)
CD3 (FITC)
non B non mono
33.8
CD14CD19 (Pacific Blue)
CD3(FITC)
b
Unstimulated
+ IL23
+ IL12
0.28
1.05
4.1
HC NK cells
0.16
0.11
1.63
Patient NK cells
0.13
0.44
1.49
HC iNKT cells
0
0
0.29
Patient iNKT cells
IFN-γ (APC-Cy7)
FSC-A
c
% IFN-γ+ (from iNKT cells)
ns
**
Unstim
IL-23
IL-12
% IFN-γ+ (from NK cells)
***
**
Unstim
IL-23
IL-12
HCs
Patient

FigS9

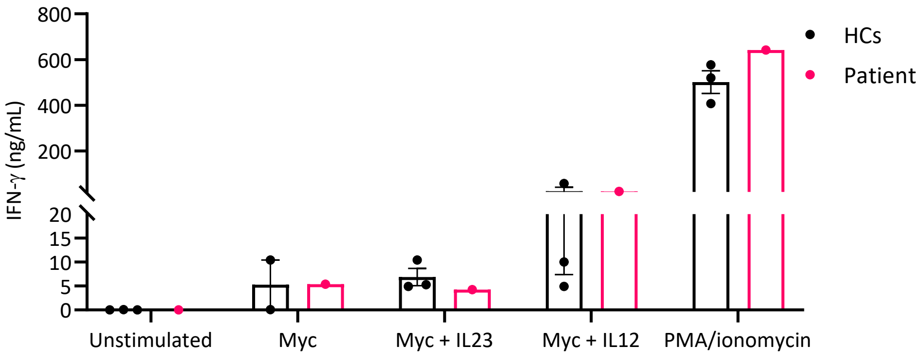

800
600
400
200
20
15
10
5
0
IFN-γ (ng/mL)
HCs
Patient
Unstimulated
Myc
Myc + IL23
Myc + IL12
PMA/ionomycin

FigS10
